# Modulation of translational decoding by m^6^A modification of mRNA

**DOI:** 10.1038/s41467-023-40422-7

**Published:** 2023-08-08

**Authors:** Sakshi Jain, Lukasz Koziej, Panagiotis Poulis, Igor Kaczmarczyk, Monika Gaik, Michal Rawski, Namit Ranjan, Sebastian Glatt, Marina V. Rodnina

**Affiliations:** 1 Max Planck Institute for Multidisciplinary Sciences, Göttingen, 37077 Germany; 2 Malopolska Centre of Biotechnology, Jagiellonian University, Krakow, 30-387 Poland; 3 Doctoral School of Exact and Natural Sciences, Jagiellonian University, Krakow, 30-387 Poland; 4 National Synchrotron Radiation Centre SOLARIS, Jagiellonian University, Krakow, 30-387 Poland

**Keywords:** Ribosome, Cryoelectron microscopy, RNA

## Abstract

N^6^-methyladenosine (m^6^A) is an abundant, dynamic mRNA modification that regulates key steps of cellular mRNA metabolism. m^6^A in the mRNA coding regions inhibits translation elongation. Here, we show how m^6^A modulates decoding in the bacterial translation system using a combination of rapid kinetics, smFRET and single-particle cryo-EM. We show that, while the modification does not impair the initial binding of aminoacyl-tRNA to the ribosome, in the presence of m^6^A fewer ribosomes complete the decoding process due to the lower stability of the complexes and enhanced tRNA drop-off. The mRNA codon adopts a π-stacked codon conformation that is remodeled upon aminoacyl-tRNA binding. m^6^A does not exclude canonical codon-anticodon geometry, but favors alternative more dynamic conformations that are rejected by the ribosome. These results highlight how modifications outside the Watson-Crick edge can still interfere with codon-anticodon base pairing and complex recognition by the ribosome, thereby modulating the translational efficiency of modified mRNAs.

## Abstract (web summary)

m^6^A is an mRNA modification that slows down translation elongation. Here, Jain et al. show that m^6^A delays decoding and increases tRNA drop-off from the ribosome by favoring alternative codon conformations that are rejected by the ribosome.

## Introduction

Post-transcriptional modifications of messenger RNA (mRNA) modulate key steps in mRNA metabolism, including mRNA stability and localization^1^, nuclear export^2^, exon-intron architecture^3^, and alternative splicing^4^. N^6^-methyladenosine (m^6^A) is one of the most abundant internal mRNA modification^5^ found in both coding and untranslated regions of mRNAs in all three domains of life with an average of three m^6^A per mRNA transcript^6,7^. The modification sites are located within a conserved consensus sequence of RRACH (R = A/G, H = U/A/C; UGCCAG in *Escherichia coli*), recognized by dedicated m^6^A methytransferases (writers) and demethylases (erasers)^8,9^. The activity of the catalytic subunit m^6^A-METTL writer complex (MAC) is stimulated by the regulatory subunit m^6^A-METTL-associated complex (MACOM)^10^. Reader proteins (e.g. eIF3, YTH domain proteins, HNRNP proteins in eukaryotes) recognize m^6^A and its consensus sequence, imparting a highly localized and regulated action^11,12^. The prevalence and conservation of m^6^A modification and of the machinery that installs, reads, and removes the modification suggests its important role in regulating mRNA dynamics and gene expression at the posttranscriptional level. Indeed, recent studies link m^6^A modification to a wide range of important biological processes such as neural development and differentiation^13^, spermatogenesis^14^, cell growth and cancer^15^. However, in most cases the exact regulatory pathway is unknown.

The majority of m^6^A sites are mapped within mRNA coding regions^5,6,16^, where the modification can modulate codon reading by aminoacyl-tRNA (aa-tRNA) in the A site of the ribosome. Most of the work on the mechanism by which m^6^A affects decoding has been carried out in the *E. coli* translation system; given the evolutionary conservation of the decoding mechanism^17,18^, similar mechanisms are likely to operate in bacteria and eukaryotes. Although the modification is not located on the Watson–Crick edge of the nucleotide, it decreases translation efficiency^19^, slows down the decoding process^20^, and alters the ribosome selectivity for the cognate aa-tRNA (i.e. fully matching the codon)^20,21^. Single-molecule experiments using Förster Resonance Energy Transfer (smFRET) that measure the duration of the decoding step (up to and including the peptide bond formation step) and tRNA–mRNA translocation showed that decoding is slowed down by m^6^A modification at the first position of the AAA codon (m^6^AAA) (15-fold) and Am^6^AA (8-fold) and to a smaller extent by AAm^6^A, Cm^6^AG and CCm^6^A modification (2.5-fold)^20^. Rapid kinetics measurements of GTP hydrolysis suggested that m^6^A reduced the initial selection capacity of the ribosome^20,21^. In addition, m^6^AAA caused a 1.5-fold excess hydrolysis of GTP per peptidyl transfer reaction, indicating that m^6^A induces excessive proofreading of cognate aa-tRNA^20^. Extrapolations of the *k*_cat_/*K*_M_ values led the authors to suggest that m^6^A dramatically (10-fold) decreases the association rate (*k*_*a*_) of the ternary complex EF-Tu–GTP–aa-tRNA (TC) to the ribosome prior to codon recognition^21^. The postulated effect would imply that the modification induces a conformational state of the ribosome that is refractory to TC binding; however, structural data in support of this hypothesis is lacking. Thus, while experiments clearly show that the m^6^A modification has an effect on translation, the exact mechanism of how m^6^A regulates individual steps of the decoding cycle remain elusive.

In this study, we systematically analyze the effect of m^6^A at all three AAA codon positions on different steps of the decoding cycle by Lys-tRNA^Lys^ using a combination of ensemble kinetics, smFRET, and single-particle cryo-EM techniques. In contrast to previous suggestions, we show that the rates of forward reactions of initial binding and codon recognition are largely unaffected, whereas the respective dissociation steps are faster in the presence of the modification, which explains the lower effective rate of GTP hydrolysis. Furthermore, the presence of m^6^A increases the transition time from codon recognition to post-decoding phase, which—together with an increased tRNA drop-off – results in a significantly fewer ribosomes reaching the translocation step. We show that the m^6^A effect depends on the position, the nature of the codon and on the chemical modifications of the tRNA anticodon. High-resolution cryo-EM structures reveal how the ribosome accommodates m^6^A modifications at different codon positions and suggest that the AAA codon adopts a structured conformation in the A site independently of the modification. In summary, the most dramatic effect of m^6^A modification appears to involve destabilization of the codon-anticodon interaction, which results in tRNA drop-off at all stages of decoding and explains the inhibitory effect of the modification on translation.

## Results

### Effect of m^6^A on the elemental steps of decoding

**Fig. 1:**
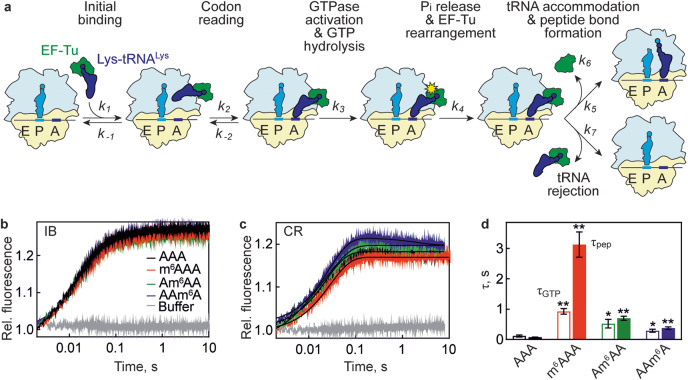
Effect of m^6^A modification on the kinetics of decoding. **a** Kinetic scheme of decoding. SSU, yellow; LSU, light blue. E, P, and A indicate the tRNA binding sites of the ribosome^22,27^. **b** Fluorescence change reporting codon-independent initial binding (IB) of TC to the ribosome. The A-site codon AAA is unmodified (black), m^6^AAA (red), Am^6^AA (green) or AAm^6^A (blue). Each time course is an average of 5-6 technical replicates. The *k*_app_ values from exponential fitting are 70 ± 1 s^−1^ (AAA), 75 ± 1 s^−1^ (m^6^AAA), 65 ± 1 s^−1^ (Am^6^AA), and 61 ± 1 s^−1^ (AAm^6^A) for the major phase (80% of the fluorescence change). Control was obtained by mixing TC with buffer (gray). **c** Fluorescence change of EF-Tu–GTP–[^14^C]Lys-tRNA^Lys^(Prf16/17) reporting A-site codon recognition (CR). Each time course is an average of 5-6 technical replicates. The *k*_*app*_ values of the major reaction (fluorescence increase) are 33 ± 1 s^−1^ (AAA), 25 ± 1 s^−1^ (m^6^AAA), 32 ± 1 s^−1^ (Am^6^AA), and 37 ± 1 s^−1^ (AAm^6^A). Controls were obtained by mixing TC with buffer (gray). **d** Reaction times (τ) of GTP hydrolysis (*τ*_GTP_, open bars) and peptide bond formation (*τ*_pep_, closed bars) from exponential fitting of the respective time courses (Supplementary Fig. 1a). Error bars indicate SEM of the fit generated from three independent experiments (N = 3). *P* values are calculated using two-tailed unpaired Student’s *t*-test in comparison to unmodified AAA codon (*P < 0.05, **P < 0.005). For *τ*_GTP,_ *P* = 0.0011 for m^6^AAA, *P* = 0.0496 for Am^6^AA and *P* = 0.0419 for AAm^6^A. For *τ*_pep_, *P* = 0.0019 for m^6^AAA, *P* = 0.0008 for Am^6^AA and *P* = 0.0023 for AAm^6^A. For all panels of Fig. 1, TC (Lys or Phe) (0.3 µM) was mixed with IC (0.9 µM). Source data are provided as a Source Data file.

We monitored the consecutive decoding steps of an AAA codon modified at any of the three codon position by its cognate Lys-tRNA^Lys^ using a fully-reconstituted in vitro translation system from *E. coli*^22^. Using the AAA codon enables to monitor the codon position effect of m^6^A within a single codon. In the first step of decoding (Fig. 1a), the ternary complex EF-Tu–GTP–Lys-tRNA^Lys^ (TC) binds to the ribosome in a codon-independent manner (characterized by the rate constants *k*_1_ and *k*_−1_), thereby initiating codon reading^17^. In the next step, codon recognition (*k*_2_ and *k*_−2_) induces domain closure of the SSU and triggers GTPase activation of EF-Tu (*k*_3_) upon EF-Tu docking at the GTPase-activating center in the large ribosomal subunit (LSU), resulting in GTP hydrolysis (*k*_GTP_)^17,23–25^. The release of the reaction product P_i_ (*k*_4_;^26^) induces the transition of EF-Tu from the GTP- to the GDP-bound conformation, allowing aa-tRNA accommodation in the A site of the LSU (*k*_5_) and peptide bond formation (*k*_pep_), whereas EF-Tu is released from the ribosome (*k*_6_). Alternatively, aa-tRNA can by rejected from the ribosome (*k*_7_)^17^. Additional rejection steps, for example after GTP hydrolysis but prior to dissociation of aa-tRNA from EF-Tu are likely^24^, but are accounted for in the global rejection rate *k*_7_. Notably, all rejection rates are very low for the cognate Lys-tRNA^Lys^ (^22^), which results in the efficient decoding and peptide bond formation.

To quantify the m^6^A effect, we prepared ribosome initiation complexes (IC) with f[^3^H]Met-tRNA^fMet^ bound to the AUG codon in the P site and an AAA (or m^6^AAA, Am^6^AA, or AAm^6^A) codon in the A site. The IC was mixed with TC that contained reporters suitable to monitor each step of decoding using ensemble rapid kinetics^22,27^. To test the effect of m^6^A modification on the initial binding step prior to codon recognition, we used an established assay with a fluorescence reporter group proflavin attached to the tRNA^Phe^ elbow region (position 16/17)^28^. The reaction does not proceed beyond initial binding, because tRNA^Phe^ (anticodon AAG) does not match the AAA codon in the A site. Interaction of EF-Tu–GTP–Phe-tRNA^Phe^(Prf16/17) with the IC leads to a rapid fluorescence increase that is largely independent of the mRNA modification, with the apparent rate constant *k*_app1_ in the range of 60–75 s^−1^ and very similar endpoints for all four codons (Fig. 1b). Although the apparent rate constant includes both the association (*k*_1_[IC]) and dissociation (*k*_*−1*_) terms, these results exclude the possibility that the association rate constant (*k*_1_ or *k*_a_) is decreased 10-fold by m^6^A modification as previously suggested^21^. Rather, the effect of the modification on the initial binding is small, if any.

Next, we monitored the codon recognition step using Lys-tRNA^Lys^ labeled by proflavin at positions 16/17 (tRNA^Lys^(Prf16/17))^22^. After initial binding to the IC, Lys-tRNA^Lys^ recognizes its cognate AAA codon, leading to a fluorescence increase when EF-Tu–GTP–[^14^C]Lys-tRNA^Lys^(Prf16/17) is mixed with IC (Fig. 1c). The apparent rate of codon recognition (*k*_app2_) is essentially independent of the m^6^A modification, showing only a small variations from approximately 33 s^−1^ with AAA to 25-37 s^−1^ with m^6^A-containing codons and a minor effect on the amplitude of the signal change, consistent with a notion that the effect of m^6^A on initial binding and codon recognition is small. In contrast, GTP hydrolysis and peptide bond formation are strongly affected (shown as reaction times *τ*_GTP_ and *τ*_pep_ for better comparison; Fig. 1d and Supplementary Fig. 1a), in agreement with previous reports^20^. With m^6^AAA codon, *τ*_GTP_ was 8-fold and *τ*_pep_ was 40-fold higher than with AAA, indicating a significant slowing down of the decoding step. The effects of modifications at the 2nd and 3rd position were somewhat lower, decreasing *τ*_GTP_ by about 5-fold (Am^6^AA) or 2.4-fold (AAm^6^A) and *τ*_pep_ by 10-fold (Am^6^AA) or 5-fold (AAm^6^A) compared to unmodified AAA. We also tested the effect of the m^6^A modification on translocation using tripeptide formation as a proxy for successful movement of the ribosome along the mRNA (Supplementary Fig. 1b). Ribosome complexes that completed decoding and contain tRNA^fMet^ in the P site and f[^3^H]Met-[^14^C]Lys-tRNA^Lys^ in the A site were mixed with EF-G to induce mRNA–tRNA translocation and a TC with Phe-tRNA^Phe^ that binds to the next codon resulting in synthesis of f[^3^H]Met-[^14^C]Lys-Phe (MKF) peptide. Comparison of the time courses of MKF synthesis shows that m^6^A at the 1st codon position slows down translocation by about 2-fold, whereas the 2nd- and 3rd-position modifications have no significant effect (Supplementary Fig. 1b).

### Context effects

**Fig. 2:**
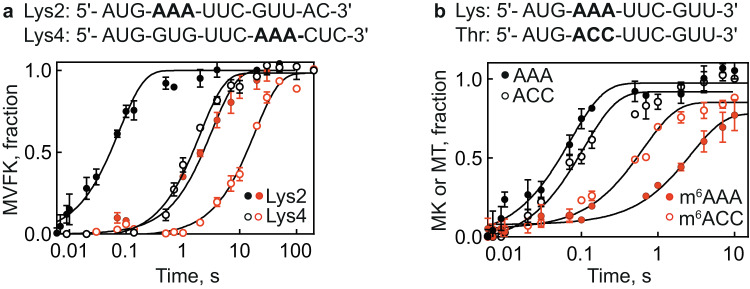
m^6^A position in the mRNA and codon identity modulates extent of translation delay. The coding sequences show the unmodified and first-position modified A-site AAA or ACC codon (bold). **a** Time courses of f[^3^H]Met-Val-Phe-[^14^C]Lys (Lys4 mRNA, open circles) and f[^3^H]Met-[^14^C]Lys (Lys2 mRNA, closed circles) formation obtained by mixing IC (0.9 μM) with the AAA (black) or m^6^AAA (red) with TC (0.3 μM) containing Val-tRNA^Val^, Phe-tRNA^Phe^, and [^14^C]Lys-tRNA^Lys^ and EF-G (4 μM) or [^14^C]Lys-tRNA^Lys^ respectively. Smooth black lines represent one-exponential fits. The reaction time τ calculated from exponential fitting is 0.08 s for AAA vs 3.3 s for m^6^AAA at Lys2 and 2 s for AAA vs 18 s for m^6^AAA at Lys4. Thus, the delay in decoding due to the modification is ~3 s at Lys2 and ~16 s for Lys4. **b** Dipeptide formation with Lys TC or Thr TC (0.3 μM) upon binding to IC (0.9 μM). f[^3^H]Met-[^14^C]Lys (closed circles) or f[^3^H]Met-[^14^C]Thr (open circles) dipeptides were separated by HPLC and quantified by scintillation counting. For both panels of this figure, each time course is the mean of three independent experiments with error bars representing standard deviation (N = 3). Source data are provided as a Source Data file.

To test whether the codon position in the mRNA attenuates the m^6^A effect, we compared the rates of AAA and m^6^AAA decoding at the 2nd (Lys2) and 4th (Lys4) positions in the mRNA. In the latter case, we translated a 4-codon sequence for Met-Val-Phe-Lys (MVFK) with unmodified AAA or m^6^AAA codon. We observe a 10-fold decrease in the *k*_pep_ value of MVFK formation with m^6^AAA relative to AAA codon, compared to the 40-fold effect observed when the codons are in the 2nd position (Fig. 2a). The m^6^A effect on Lys4 is very similar to that observed by measuring ribosome rotation (15-fold)^20^, which is a proxy for peptide bond formation. However, taking into account that incorporation of Lys4 is slower than of Lys2 due to additional time needed for MVF synthesis, the actual delay in decoding of the modified Lys4 codon (approximately 16 s; Fig. 2a) is even longer than on Lys2 (3 s; Fig. 1d). These results suggest that the m^6^A effect can be attenuated by the mRNA context, in agreement with the previous results^20^.

m^6^A causes translation delays not only on modified AAA, but also on Cm^6^AG (coding for Gln) and CCm^6^A (Pro) codons^20^, whereas the effect on the ACC (Thr) codon was not tested. Notably, ACC is part of the RRACH consensus sequence that is likely to be methylated in vivo. We measured the effect of m^6^A on the kinetics of dipeptide formation (MK or MT) with IC programmed by the respective mRNAs and TC (Lys or Thr). Our results show that the effect on the ACC codon is smaller than on AAA, 5-fold vs. 40-fold, respectively (Fig. 2b). Thus, also the codon/amino acid identity modulates the translation delay caused by the m^6^A modification.

### Interplay with tRNA modifications

**Fig. 3:**
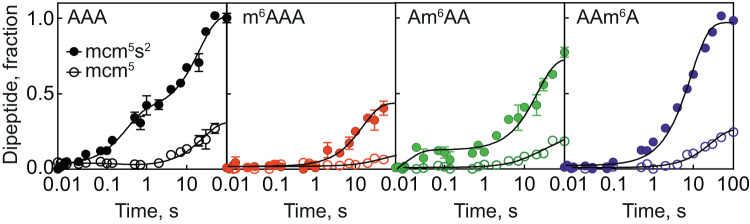
Interplay between mRNA and tRNA modifications. Time courses of fMet-[^14^C]Lys formation obtained by mixing TC containing yeast [^14^C]Lys-tRNA^Lys^ (0.3 μM) with IC (0.9 μM). [^14^C]Lys-tRNA^Lys^ is fully modified with mcm^5^s^2^U_34_ (closed circles) or hypomodified containing mcm^5^U_34_ (open circles). Each curve is the mean of three independent experiments with error bars representing standard deviation (*N* = 3). Smooth black lines represent single or double exponential fits. fMet-[^14^C]Lys dipeptides were separated by HPLC and quantified by scintillation counting. The *τ*_pep_ values of the fast reaction with fully modified mcm^5^s^2^U_34_ [^14^C]Lys-tRNA^Lys^ are approximately 10–20 s^−1^ irrespective of the modification, the reactions with mcm^5^U_34_ tRNA are too inefficient to obtain reliable values. Source data are provided as a Source Data file.

In the cell, not only mRNA, but also tRNAs can be modified, which prompted us to test how modifications at the tRNA anticodon affect reading of m^6^A-modified codons. We made use of the known detailed kinetic mechanism of AAA decoding by yeast tRNA^Lys^ containing mcm^5^s^2^ modification at U_34_ and the well-documented contribution of the s^2^U_34_ to decoding^22^. The mcm^5^s^2^ modification at U_34_ is introduced by the *ELP*^29^ and *URM1* pathways^30^. Deletion of the *URM1* gene in yeast results in synthesis of Δs^2^U_34_ tRNA^Lys^, which carries all other modifications but lacks s^2^U_34_^31^, as verified by ((N-acrylolamino)phenyl)mercuric chloride (APM) gel retardation of tRNA (Methods). The hypomodified tRNA shows an increased propensity to dissociate from the ribosome (manifested in the increased *k*_*−2*_ and *k*_*7*_ dissociation rate constants), slower rearrangement after GTP hydrolysis (*k*_*4*_) and slower accommodation into the peptidyl transferase center (*k*_*5*_)^22^. To test how a fully modified mcm^5^s^2^ Lys-tRNA^Lys^ and Δs^2^U_34_ Lys-tRNA^Lys^ affect m^6^A decoding, we monitored fMet-[^14^C]Lys formation with IC containing a unmodified or m^6^-modified AAA codon in the A site (Fig. 3). The time course of peptide bond formation with the fully modified mcm^5^s^2^ Lys-tRNA^Lys^ on the AAA codon is biphasic (Fig. 3a), which reflects heterogeneous pathways towards aa-tRNA accommodation depending on the timing of its release from EF-Tu (*k*_5a_ and *k*_5b_, respectively)^22^. Removal of the s^2^U_34_ modification dramatically reduces the rate and end level of the reaction (Fig. 3), in agreement with the earlier report^22^. With m^6^A modification at any codon position, the rate of peptide bond formation with mcm^5^s^2^ Lys-tRNA^Lys^ is very low, 30–80-fold lower than on an unmodified AAA codon (Fig. 3). Furthermore, the end level is reduced, indicating that a large fraction of Lys-tRNA^Lys^ dissociates from the ribosome before entering the reaction. The effect is even more dramatic when the m^6^A modification on the mRNA is combined with the Δs^2^U_34_ Lys-tRNA^Lys^, in particular on m^6^AAA and Am^6^AA codons. These data suggest that m^6^A modification enhances rejection of mcm^5^s^2^ Lys-tRNA^Lys^ and the lack of s^2^U_34_ tRNA modification further destabilizes the complexes, thereby practically abolishing translation.

### The mechanism of decoding inhibition by m^6^A

**Fig. 4:**
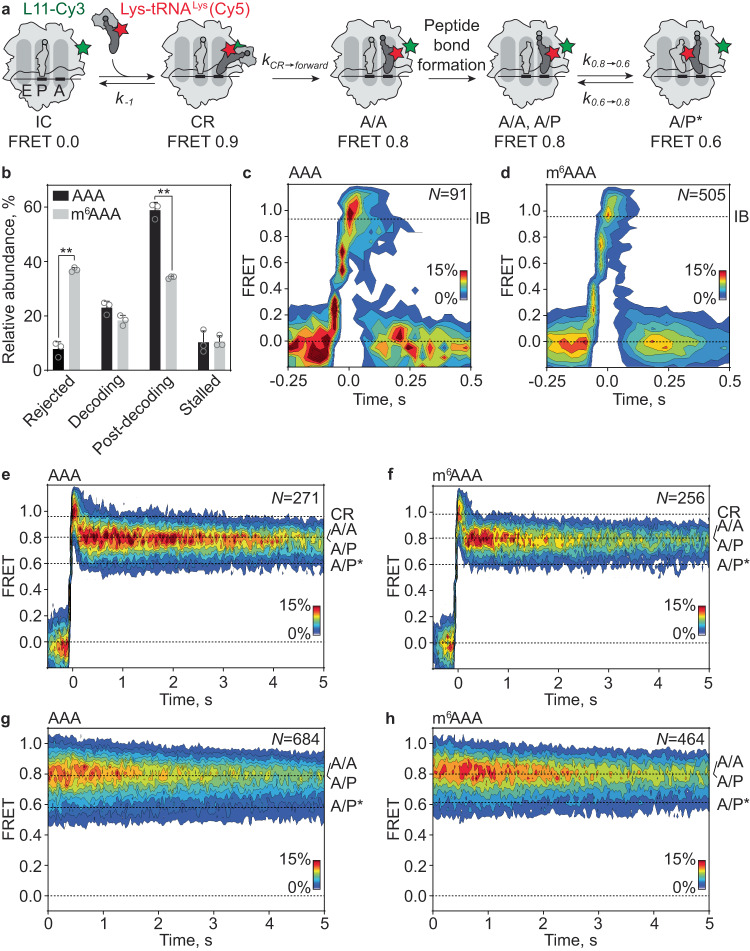
Decoding of AAA and m^6^AAA monitored by smFRET. **a** Schematic of the smFRET experiment. 70S IC (light gray) is labeled with Cy3 (green star) at the protein L11 and Lys-tRNA^Lys^ is labeled with Cy5 (red star). **b** Relative abundance of the different types of traces during decoding of AAA (black) or m^6^AAA (gray) codon. “Rejected” denotes the fraction of complexes that dissociate from the ribosome after initial binding (IB; **c**, **d**). “Decoding” pertains to traces that show the characteristic codon reading (CR) phase followed by accommodation and ensuing fluctuations between A/A, A/P, and A/P* states (**e**, **f**). “Post-decoding” are the complexes in which Lys-tRNA^Lys^ is found in an A/A, A/P or A/P* state at the start of the recording (**g**, **h**). “Stalled” denotes the fraction of ribosomes that do not progress past the CR state (Supplementary Fig. 2). Shown are the mean values with error bars representing standard deviation from *N* = 3 independent experiments. ** indicates statistical significance with *P* < 0.01 using a two-tailed Welch’s t-test without adjustment for multiple comparisons. *P* = 0.0015 for rejected traces, *P* = 0.0038 for post-decoding traces, *P* = 0.0565 for decoding traces. Open circles are the values in the individual experiments. **c** Contour plot showing the distribution of FRET values after synchronization during initial binding and dissociation of Lys-tRNA^Lys^(Cy5) from ICs carrying the AAA codon in the A site. Traces are synchronized to the first transition with FRET > 0. *N*, number of traces. **d** same as **c** on the m^6^AAA codon. **e** Contour plot showing the distribution of FRET values during the full decoding trajectory on the AAA codon. FRET 0.96 ± 0.03 in the CR state derived from mean ± s.d. of the µ values of the Gaussian fitting of N = 3 independent experiments. **f** same as **e** on the m^6^AAA codon, FRET 0.98 ± 0.01. **g** Contour plot showing the distribution of FRET values in the post-decoding complexes carrying fMet-Lys-tRNA^Lys^(Cy5) on the AAA codon. FRET population distribution reveals two states with FRET 0.79 ± 0.01 (A/A, A/P) and 0.58 ± 0.02 (A/P*)^35,64–66^. **h** Same as **g** on m^6^AAA codon. A/A, A/P, FRET 0.80 ± 0.01; A/P*, FRET 0.61 ± 0.01. Data are from N = 3 independent experiments. Source data are provided as a Source Data file.

While there is a clear effect of m^6^ modification on GTP hydrolysis and dipeptide formation, the model explaining these effects by a slower association rate of the TC to the ribosome^21^ is clearly not supported by our experimental data. This prompted us to examine the mechanism of decoding in more detail using smFRET approaches^32–35^ comparing AAA and m^6^AAA codons, which gave the largest rate differences. smFRET experiments are designed to monitor binding and unbinding of TC to and from the ribosome, as well as movement of aa-tRNA through the ribosome, accommodation and the dynamics of the peptidyl-tRNA after peptide bond formation^32,33^. TC binding to the ribosome was monitored by smFRET between the ribosomal protein L11 labeled with FRET donor Cy3 (L11-Cy3) and Lys-tRNA^Lys^ labeled with FRET acceptor Cy5 (Lys-tRNA^Lys^(Cy5)) (Fig. 4a). L11-Cy3 IC was immobilized on coverslips through 5′-biotinylated mRNAs carrying AAA or m^6^AAA codons in the A site. After injection of TC with Lys-tRNA^Lys^(Cy5) into the flow chamber, we observed four types of traces. The percentages of ribosomes following each of type of trajectories are strikingly different for AAA and m^6^AAA (Fig. 4b). Traces that show rapid (>30 s^−1^) appearance of FRET signal followed by a rapid loss of FRET reflect initial binding complex formation and dissociation prior to codon recognition (Fig. 4b–d and Supplementary Fig. 2a). Such traces comprise only a small percentage of complexes on the AAA codon (8 ± 2%), but increase to 37 ± 1% (*P* = 0.0015) on m^6^AAA. The dissociation rate (*k*_−1_) of these traces is higher with m^6^AAA, indicating that m^6^A promotes rejection of ternary complexes from the initial binding complex (Fig. 4b, Supplementary Fig. 2b and Table 1).

**Table 1:** Kinetic analysis of decoding of AAA and m^6^AAA codons

	AAA	m^6^AAA
*k*_*−1*_^a^, s^−1^, (*n*)^b^	8.1^c^ (90)	15 ± 1^d^ (495)
*k*_*CR→forward*_, s^−1^ (*n*)	6.3 ± 0.7 (261)	7.2 ± 2.2 (256)
*k*_*−2*_^e^, s^−1^, (*n*)	0.34 ± 0.02 (161)^f^	1.1 ± 0.1 (95)^f^
*k*_CR→A/P*_, s^−1^ (*n*)	2.0 ± 0.9 (138)	1.0 ± 0.6 (125)
*k*_0.8→0.6_, s^−1^ (*n*)	2.9 ± 0.6 (433)	2.8 ± 0.2 (710)
*k*_0.6→0.8_, s^−1^ (*n*)	4.4 ± 0.4 (441)	4.2 ± 0.2 (701)

^a^Shown are the corrected rates *k*_corrected_ according to *k*_corrected_*= k*_observed_ *− k*_photobleach_ *− 1/T*, where *k*_observed_ the rate of the single-exponential decay function, *k*_photobleach_ = 0.03 ± 0.01 s^−1^ and *T* the observation window, 33 s^35^.

^b^*n*, total number of transitions from three independent experiments.

^c^Number of traces is too small to calculate the rate in the independent datasets.

^d^Shown are the mean and standard deviation from 3 independent experiments.

^e^From the experiment with EF-Tu(H84A).

^f^The difference in the rates is statistically significant (*P* = 0.0052).

Two other types of traces correspond to ribosome complexes where tRNA is successfully accommodated in the A site. In those traces that show a full decoding trajectory, rapid (>30 s^−1^) codon reading results in appearance of high FRET 0.9, followed by the progression to lower FRET states (step assignment is from^33^). The latter corresponds to tRNA movement from codon reading towards the accommodation in the A site and peptide bond formation. After peptide bond formation, fMet-Lys-tRNA^Lys^(Cy5) starts to fluctuate between A/A, A/P (FRET 0.8) and A/P* (FRET 0.6) states (Fig. 4e, f and Supplementary Fig. 2c). The percentage and the decay rate of the codon reading state (*k*_CR→forward_) are not strongly affected by the m^6^ modification (Fig. 4b, Supplementary Fig. 2d and Table 1), suggesting that the codon reading state does not accumulate during the reaction. However, the transition rate *k*_CR→A/P*_ from the codon reading (FRET 0.9) to the post-decoding A/P^*^ state (FRET 0.6) is reduced 2-fold in the presence of m^6^A (Supplementary Fig. 2c, d and Table 1).

A significant fraction of ribosome complexes reached the post-decoding state already at the starting point of imaging, with fMet-Lys-tRNA^Lys^(Cy5) fluctuating between FRET 0.8 (A/A, A/P) and 0.6 (A/P*) states (Fig. 4g, h and Supplementary Fig. 2e). Here, we observe no significant differences in the transition rates between A/A, A/P (FRET 0.8) and A/P* (FRET 0.6) states (*k*_0.8→0.6_ and *k*_0.6→0.8_) in the presence of m^6^A, indicating that m^6^A has no effect on the tRNA^Lys^ dynamics in classical and hybrid states (Supplementary Fig. 2f and Table 1). However, the percentage of ribosomes showing classical to hybrid fluctuations is reduced in the presence of m^6^A (*P* = 0.0038, Fig. 4b), consistent with the notion that a fraction of ribosomes has lost the tRNA before entering the post-decoding phase. Lastly, we also observed a small fraction of traces that did not progress beyond the codon reading state, but the percentage of these traces was not affected by the presence of m^6^A (Fig. 4b and Supplementary Fig. 2g–i). In conclusion, m^6^A increases the dissociation of TC during initial binding and codon reading, as well as delays the transition to the post-decoding state after GTP hydrolysis.

**Fig. 5:**
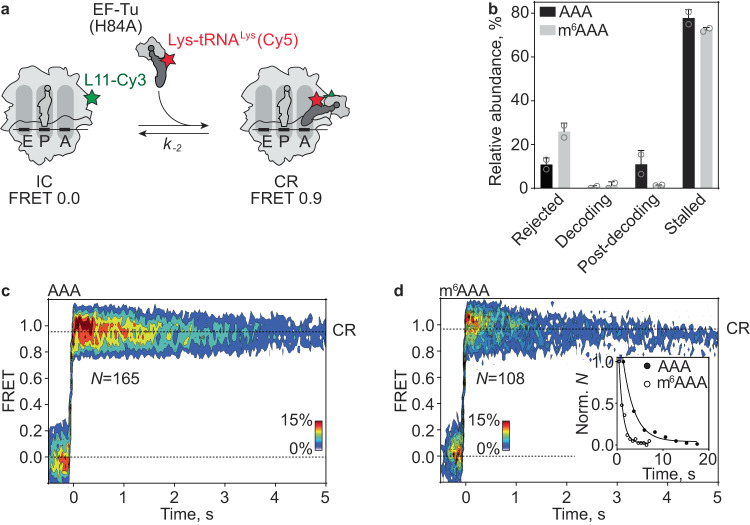
Codon reading of AAA and m^6^AAA by EF-Tu(H84A)–GTP–Lys-tRNA^Lys^(Cy5). **a** Schematic of the smFRET experiment monitoring decoding attempts by the GTPase-deficient TC. CR, codon reading. **b** Relative abundance of smFRET traces during decoding of AAA (black) or m^6^AAA (gray) codons by EF-Tu(H84A). Shown are the mean values and error bars represent standard deviation from N = 2 independent experiments. Open circles are the values in the individual experiments. **c** Contour plot showing the distribution of FRET values after synchronization during CR (FRET 0.97 ± 0.01) without progression to accommodation of Lys-tRNA^Lys^(Cy5) on AAA codon. Data are from N = 2 independent experiments. **d** Same as **c** for the m^6^AAA codon (FRET 0.96 ± 0.01). Inset shows normalized dwell-time histograms of EF-Tu(H84A)–GTP–Lys-tRNA^Lys^(Cy5) dissociation (*k*_−2_) during decoding of AAA (closed circles) and m^6^AAA (open circles) codon (Table 1). Normalization was performed by division by the maximum value of the histogram. Source data are provided as a Source Data file.

To further investigate the effect of m^6^A on the stability of codon-anticodon duplex in the A site, we repeated the experiments using EF-Tu(H84A), a GTPase-deficient EF-Tu mutant (Fig. 5a). Steps prior to GTP hydrolysis, i.e. initial binding, codon recognition and GTPase activation, are not affected by the mutation, but GTP hydrolysis and the subsequent steps of tRNA accommodation and peptide bond formation are abolished^36^. In the presence of EF-Tu(H84A), the TC stalled in the codon reading state represents the majority of the population (78 ± 3% for AAA and 72 ± 1% for m^6^AAA) (Fig. 5b), in agreement with previous studies using non-hydrolysable GTP analogs^20,33^. For both AAA and m^6^AAA, FRET population distribution revealed a single FRET 0.9 codon reading state that did not progress towards accommodation (Fig. 5c,d). The decay rate of the long-lived codon reading state reports the dissociation of the ternary complex from the ribosome and acts as a proxy for codon-anticodon stability^22,27^. In the presence of m^6^A, the decay rate (*k*_−2_) is 3.2-fold higher that on the unmodified AAA codon (Fig. 5d, inset), indicating that m^6^A destabilizes the codon-anticodon interactions during codon reading prior to GTP hydrolysis. This leads to rapid dissociation from the ribosome and thus further accounts for low efficiency of tRNA accommodation.

### Cryo-EM captures dipeptidyl-tRNA^Lys^ bound to m^6^A-modified codons

**Fig. 6:**
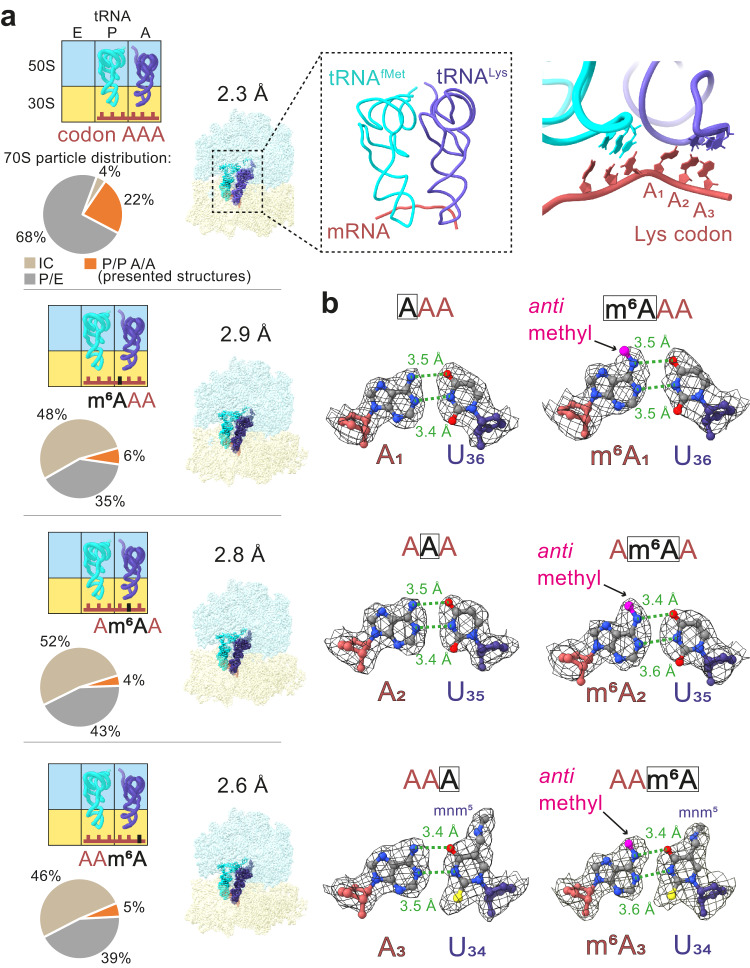
Cryo-EM analysis of ribosome complexes with m^6^A modification at different codon positions. **a** From top to bottom: AAA, m^6^AAA, Am^6^AA, AAm^6^A (top left: schematic view, right: map colored by the RNA chain. LSU is shown in light blue, SSU in yellow, mRNA in red, P-site tRNA in cyan and A-site tRNA in dark blue). The pie charts show relative particle distributions of different states. Lys codons carrying an m^6^A modification lead to a lower proportion of ribosomes progressing towards decoding (orange slice). The maps are shown as surface representation. Top middle panel, close-up view of the P-site and A-site tRNAs and mRNA. Top right panel, close-up view of mRNA–tRNA interactions. **b** Base pairing at the 1st, 2nd and 3rd codon position. Left, AAA codon; right, the respective m^6^A-modified pair. The m^6^ methyl group (magenta) is shown in an *anti* conformation. The measured distances between the conventional hydrogen bond donor and acceptor indicated with a dashed line are expressed in Å. The maps are shown as mesh with contour rmsLevel 4.

Next, we obtained the structures of ribosome complexes with tRNA^Lys^ in the A site using single-particle cryo-EM. The complexes were prepared by mixing EF-Tu–GTP–Lys-tRNA^Lys^ with IC containing fMet-tRNA^fMet^ in the P site and mRNAs with either AAA, m^6^AAA, Am^6^AA, or AAm^6^A in the A site. We collected four equivalent datasets (Supplementary Table 1) and sorted the particles according to the tRNA occupancy of the E, P, and A sites (Supplementary Fig. 3). In addition to a fraction of unreacted IC, we found ribosomes that completed the decoding process and contained tRNA^f^^Met^ in the P site and fMet-Lys-tRNA^Lys^ in the A site. The latter complexes could be further classified into (i) those where tRNAs were in classical (P/P, A/A) states with ribosomes in a non-rotated conformation and (ii) those with tRNAs in hybrid (P/E, A/P) states with the ribosomal subunit rotated relative to each other. The particle distribution was dramatically different between samples with unmodified AAA and m^6^A-modified codons (Fig. 6a). Specifically, 48% (m^6^AAA), 52% (Am^6^AA), or 46% (AAm^6^A) of ribosomes failed to progress from the IC towards decoding and peptide bond formation, compared to only 4% of ICs with unmodified AAA codon. These differences support the notion that a sizable fraction of Lys-tRNA^Lys^ that attempts to bind to its cognate codon is rejected during the decoding process.

The particles with tRNAs in classical states were used to reconstruct maps at 2.3 Å (AAA), 2.9 Å (m^6^AAA), 2.8 Å (Am^6^AA), and 2.6 Å (AAm^6^A) global resolutions (GSFSC with 0.143 cut-off; Fig. 6a), respectively. The high quality of the maps in the proximity of the decoding region (Supplementary Fig. 4) allowed the direct visualization of the m^6^A modification at different codon positions (Supplementary Fig. 5). Furthermore, the fMet-Lys moieties in the peptidyl transferase center (Supplementary Fig. 6), as well as all known tRNA^fMet^ and tRNA^Lys^ modifications (e.g. Cm_32_, mnm^5^U_34_) could be assigned. However, detailed comparisons did not reveal any pronounced structural differences between the models with and without m^6^A (Fig. 6b). Of note, we positioned the m^6^A in the *anti* conformation, which enables hydrogen bonding in all three codon-anticodon pairs. Even at the high resolution obtained, the cryo-EM densities do not unambiguously exclude the *syn* conformation. However, fitting m^6^A in a *syn* conformation precludes hydrogen bonding and results in an increased distance of the N6 atom to the O4 atom from the cognate U in the codon by ~0.5 Å (Supplementary Fig. 7). Thus, once the A-site aa-tRNA is bound, we cannot detect any major structural differences between unmodified and m^6^A-modified codons.

### The AAA codon is structured in an unoccupied A site

**Fig. 7:**
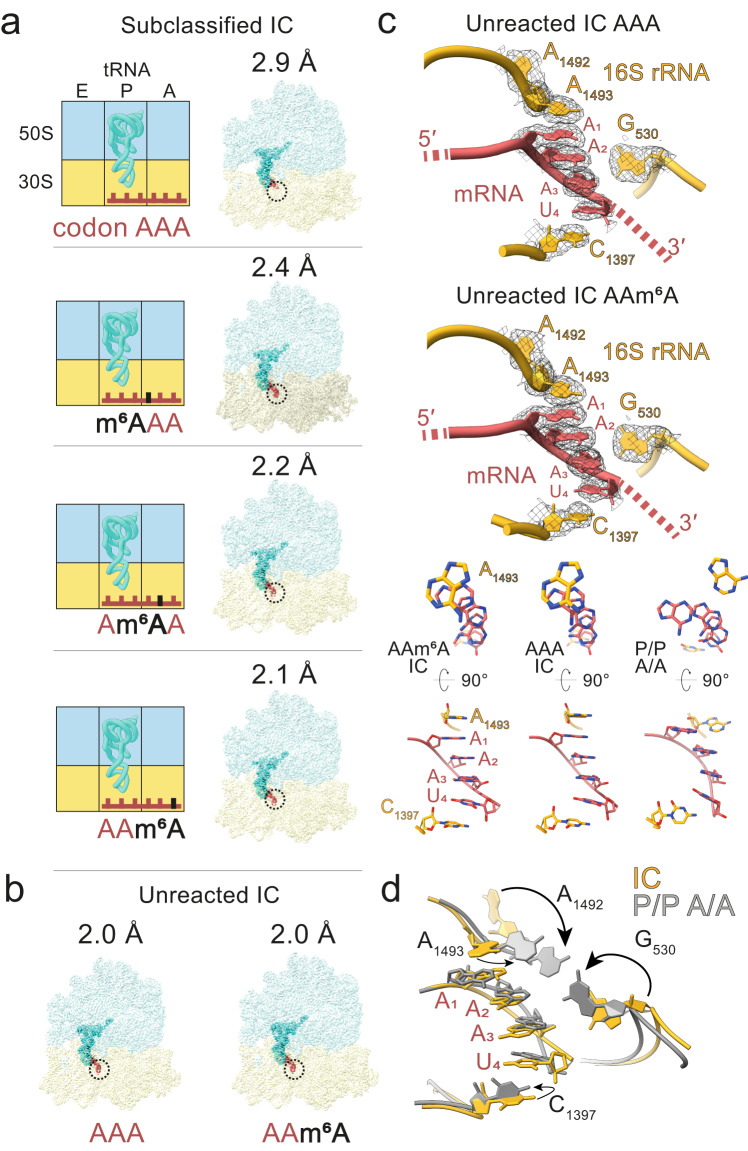
Cryo-EM structures of ICs with m^6^A in different positions of the A-site codon. **a** From top to bottom: AAA, m^6^AAA, Am^6^AA, AAm^6^A. Left panel: schematic view; right panel: map colored by the RNA chain. LSU is shown in blue, SSU in yellow, mRNA in red, P-site tRNA in cyan. The A-site codon is indicated with dashed circles. **b** Structures of unreacted ICs (i.e. prepared without addition of Lys-tRNA^Lys^, see text) with AAA mRNA (left) and Am^6^AA mRNA (right). Maps are shown in surface representation. **c** Close-up view of interactions between AAA or AAm^6^A A-site codon (red), and rRNA (yellow) in the decoding region of the unreacted ICs (top). The corresponding models are also shown from different perspectives (bottom). In the unreacted AAA (left) and AAm^6^A ICs (middle) the nucleotides form a π-stacking array, whereas the equivalent nucleotides in the post-decoding complex (P/P A/A) appear unstacked (right). **d** Repositioning of the 16S rRNA nucleotides A1493, A1492, G530 and C1397. The conformational rearrangements during the transition from IC (yellow) to the post-decoding complex (P/P A/A; gray) are shown in reference to the mRNA codon.

To further elucidate the structural basis of slower translational rates of ribosomes decoding the m^6^A-modified Lys codon, we separately analyzed ICs from the same datasets and reconstructed maps at 2.4 Å (m^6^AAA), 2.2 Å (Am^6^AA), and 2.1 Å (AAm^6^A) overall resolution (Supplementary Fig. 8). Because the dataset for the IC with unmodified AAA codon was too small to obtain a structure at a similar resolution range (Fig. 7a), we collected two additional IC datasets with either an AAA or AAm^6^A codon in the A site (Fig. 7b). The complexes were prepared in the same way as described above, but without adding Lys-tRNA^Lys^; the final overall resolution of both structures was 2.0 Å (Supplementary Fig. 9 and 10). Despite the high quality of the maps, particularly in the decoding region (with local resolution between 2.4–2.7 Å), the density for the m^6^ modification was not well resolved (Supplementary Fig. 11). Surprisingly, in all structures the AAA codon was in a π-stacking conformation (Fig. 7c), which—to our knowledge—was not identified with any other codon sequence in the well-resolved IC structures available so far. The π-stacking was stabilized by the interactions with the decoding center of the ribosome, in particular 16S rRNA nucleotides A1493 and C1397. Residue A1493 in helix 44 (h44) flips out of its helical arrangement and is involved in stacking with A1 of the AAA codon. The stacking array extends beyond the A-site codon to the next nucleotide (U_4_) from the downstream codon UUC and with C1397 of 16S rRNA. Another key residue in the decoding center, A1492, remains stacked within an internal loop of h44 of the IC, whereas G530 is pushed away from the mRNA and does not appear to be engaged in the codon stabilization (Fig. 7c).

Comparison between the unpaired AAA codon in IC and the post-decoding complex with fMet-Lys-tRNA^Lys^ in the A site shows that the positioning of the 16S rRNA nucleotides C1397, A1492, A1493, and G530 changes between the two states (Fig. 7d). Upon decoding, the base array of A_1_-U_4_ mRNA becomes unstacked. The C1397 base rotates by approximately 90° with respect to the U_4_. While, in the IC, A1493 is involved in the stabilization of the A-site codon, in the decoding complex, A1492, A1493, and G530 monitor the correct Watson–Crick geometry of the codon-anticodon complex in the A site, consistent with previous findings^37,38^. A1493 and A1492 are flipped-out of the 16S rRNA stacking conformation and together with G530 interact with the minor grove of the codon-anticodon mini helix. Of note, the positions of these rRNA and mRNA nucleotides are identical in the m^6^A-modified and the AAA datasets (Fig. 7c), showing that m^6^A does not affect monitoring of the codon-anticodon complex by the ribosome.

## Discussion

The results of this work, together with previous reports^20,21^, show that m^6^A modification on AAA and other A-containing codons, such as Glu, Gln, Pro, and Thr, leads to inefficient decoding in a codon context-dependent manner. We show that, in contrast to previous suggestions^21^, m^6^A does not affect the association rate of the TC to the ribosome and provide detailed insights into how m^6^A modifications modulate the decoding process during translation elongation. Prior to decoding, the AAA codon adopts a π-stacking arrangement involving the 3’-adjacent U in the mRNA and stabilized by A1493 and C1397 of 16S rRNA (Fig. 7). While the codon-independent initial binding of the cognate TC-Lys to the AAA-programmed ribosomes is rapid and independent of the m^6^A modification (Figs. 1 and 3), the resulting complexes tend to dissociate more rapidly when the modification is present (Table 1; Figs. 4 and 5). As a result, fewer ribosomes proceed towards rapid codon recognition (Fig. 4). The presence of m^6^A reduces the stability of the codon recognition complex (Table 1) and slows down GTP hydrolysis and accommodation compared to the unmodified codon (Fig. 1 and refs. ^20,21^). The delays in the forward decoding steps together with the increased tRNA^Lys^ drop-off from the ribosome result in fewer ribosomes that complete decoding (Figs. 4 and 6). On those ribosomes that reached the post-decoding state, the π-stacking arrangement of the codon is resolved, and the codon-anticodon complex adopts a canonical Watson-Crick geometry stabilized by the residues of 16S rRNA independent of m^6^A modification (Fig. 6).

Comparison of the structural and biophysical data provides insights into the dynamics of AAA decoding and the role of the m^6^A modification. The observed π-stacking codon arrangement is similar to that found in yeast ribosomes and is stabilized by homologous nucleotides (Supplementary Fig. 12 and refs. ^39,40^), indicating evolutionary conservation of the recognition mechanism. However, our data show that a single unmodified AAA codon is efficiently decoded despite the unfavorable codon conformation (Figs. 1 and 6), indicating that the structuring of the A-site codon alone cannot explain translational stalling on extended poly(A) tracks, as previously suggested^39,40^. Also the presence of an AAAA sequence in the yeast structure instead of an AAAU in our structures cannot explain the difference, as AAAAAA sequences are translated efficiently^39^. Concerning the effect of the m^6^A modification, previous NMR data suggested that m^6^A stabilizes stacking compared to the unmodified base^41^. A stronger codon stacking in a conformation unfavorable for codon reading may explain why a sizable fraction of TCs that scan the ribosome during the initial binding and codon reading are rapidly rejected from the ribosome prior to forming a codon-anticodon complex, as indicated by smFRET experiments (Fig. 4b–d).

The rates of the codon recognition on those ribosomes that proceed towards decoding are not much changed by the m^6^A modification (Fig. 1c). This observation favors the conformational selection model, which assumes that the π-stacking and unstacked arrangements are in dynamic equilibrium on the ribosome. If the TC attempting to read the A site codon encounters the stacked conformation, the respective attempt is likely to fail, resulting in the dissociation of the TC prior to codon recognition. In contrast, when TC encounters an unstacked conformation, it can rapidly form a codon-anticodon complex and proceed towards tRNA accommodation. At this stage, also the switch from *syn* to *anti* conformation of the m^6^ group may play a role, as the optimal codon-anticodon pairing is accomplished by rotating away from its energetically preferred *syn* geometry on the Watson-Crick face to the higher-energy *anti* conformation^41^. The m^6^ modification is poorly resolved in the cryo-EM structures with the vacant A site, despite the high quality of the 70S IC structures (Fig. 7a, b). Given that the nucleotides are clearly resolved, the weak density for the modification (Supplementary Fig. 11) likely reflects its rotational dynamics. Notably, the density for the additional methyl group is better resolved in the post-decoding complexes (Supplementary Fig. 5) indicating that tRNA binding to the codon restricts m^6^ dynamics. Fitting the density with m^6^A in *anti* conformation yielded a perfect Watson–Crick geometry of base pairing (Supplementary Fig. 7), whereas using the *syn* conformation results in a shift of the entire nucleotide. The unfavorable *anti* conformation is partially compensated by hydrogen bonding and Watson–Crick pairing with U resulting in the net destabilization by 0.5–1.7 kcal/mol for a single methyl substitution^42^. The fluctuations between *syn* and *anti* conformation of m^6^A in combination with the steric hindrance of the codon-anticodon pairing in the energetically more favorable *syn* conformation may cause the destabilization of the codon-anticodon complex observed in smFRET measurements (Table 1). The altered dynamics of the codon-anticodon complex may also affect the propensity of the SSU to form a closed global conformation that is essential for the activation of GTP hydrolysis^25,43,44^, which may provide a potential mechanistic explanation for the observed slowing down of GTP hydrolysis.

The accommodation of tRNA^Lys^ is also affected by m^6^A, in particular for the m^6^AAA codon, which is manifested by a further delay of peptide bond formation after GTP hydrolysis (Fig. 1d). smFRET experiments reveal the tendency for a delayed entry into the FRET 0.6 state upon the formation of tRNA hybrid states after peptide bond formation (Table 1). These data suggest that m^6^A enriches for conformations that enhance tRNA dissociation and disfavor the forward steps towards completion of the decoding step. However, the complexes that completed decoding are found in the canonical tRNA conformation regardless of the modification (Fig. 6). Thus, the ribosome rectifies the codon-anticodon dynamics to select the correct codon-anticodon geometry and the m^6^A modification affects this process, potentially by stabilizing otherwise unfavorable codon-anticodon conformations. Similarly, the ribosome selectively stabilizes G-U mismatches in a tautomeric form that is unfavorable in solution, but is favored by the ribosome, because it allows pairing with Watson–Crick geometry^42,45^, thus underscoring the generality of the ribosome rectification mechanism.

The dynamics of the codon-anticodon complex is further modulated by the modifications in the tRNA anticodon (Fig. 3). With yeast tRNA^Lys^, the presence of m^6^A had a strong effect not only on the rate of peptide bond formation, but also on the end level of reaction, which indicates enhanced drop-off of tRNA^Lys^ from the ribosome during decoding. Similarly to the reaction with *E. coli* tRNA^Lys^, the most dramatic effect was observed with m^6^AAA and the smallest with AAm^6^A. Removal of the s^2^ modification from yeast mcm^5^s^2^U_34_ essentially inhibits the reaction (Fig. 3), underscoring the complex interplay between the dynamics of the codon and the properties of the tRNA anticodon for the outcome of tRNA selection. Taken together, our work combining ensemble kinetics, smFRET and cryo-EM methods provides detailed mechanistic insights into how m^6^A modification modulates decoding.

## Methods

70S ribosomes, EF-Tu and f[^3^H]Met-tRNA^fMet^ from *E. coli* were prepared as described in previous studies^27,46,47^. Unmodified and m^6^A-modified mRNA was purchased from IDT (Iowa, USA). Native Lys-tRNA^Lys^, Lys-tRNA^Lys^(Prf16/17) and Phe-tRNA^Phe^ from *E. coli* and yeast Phe-tRNA^Phe^(Prf16/17) were prepared as described^22,48^. Yeast mcm^5^s^2^U_34_ and mcm^5^U_34_ Lys-tRNA^Lys^ were prepared from wild-type S288C and Δ*URM1* yeast strains, respectively^31^, and the s^2^ modification was quantified as described^22^. The extent of s^2^ modification was verified by ((N-acrylolamino)phenyl)mercuric chloride (APM) gel retardation of tRNA and was 70–90% for native tRNA^Lys^ and 2% for *urm1*Δ tRNA^Lys^. The following mRNAs (unmodified and m^6^A-modified at different codon positions of AAA or ACC codon) were purchased from IDT (Iowa, USA). The coding region (starting with AUG) is separated from the 5’UTR by space and the m^6^ residue is introduced in one of the underlined A positions.

5′-GGCAAGGAGGUAAAUA AUGAAAUUCGUUAC-3′

5′-GGCAAGGAGGUAAAUA AUGACCUUCCGCCUCUCUCUC-3′

5′-GGCAAGGAGGUAAAUA AUGGUGUUCAAACUGCGCCUCUCUCUC-3′

For the smFRET experiments, the following 5′-biotinylated mRNA (unmodified and m^6^A-modified) was used.

5′-Biotin-CAACCUAAAACUUACACACCCGGCAAGGAGGUAAAUA AUGAAAUUC AUUACCUAA-3′

EF-Tu–GTP–[^14^C]Lys-tRNA^Lys^ (Prf16/17) (TC-Lys) was prepared by incubating EF-Tu (75 µM), GTP (1 mM), PEP (3 mM), DTT (1 mM), pyruvate kinase (1% v/v), tRNA^Lys^(Prf16/17) (15 µM), ATP (3 mM), L-[^14^C]lysine (22.5 µM) and Lys-tRNA synthetase (2% v/v) in buffer A containing 50 mM Tris-HCl pH 7.5, 70 mM NH_4_Cl, 7 mM MgCl_2_ and 1 mM DTT_._ Assembled TC-Lys was purified by gel filtration on two Superdex 75 HR columns operated in tandem (GE Healthcare) in buffer A. EF-Tu–GTP–[^14^C]Phe-tRNA^Phe^(Prf16/17) and EF-Tu–[γ-^32^P]GTP–[^14^C]Lys-tRNA^Lys^ were prepared and purified in similar ways.

IC was prepared from 70S ribosomes (2 µM), mRNAs as indicated (6 µM), f[^3^H]Met-tRNA^fMet^ (3 µM), IF1, IF2, IF3 (3 µM each), DTT (1 mM) and GTP (1 mM) in buffer TAKM_7_ (50 mM Tris-HCl pH 7.5, 30 mM KCl, 70 mM NH_4_Cl and 7 mM MgCl_2_) for 30 min at 37 °C. IC was purified by centrifugation through 1.1 M sucrose cushion in TAKM_7_ for 2 h at 4 °C and 259,000 × *g* in Beckman Optima Max-XP ultracentrifuge. After centrifugation, the pellets were dissolved in TAKM_7_ and quantified by scintillation counting.

### Rapid kinetics

All fluorescence stopped-flow experiments were performed in buffer A at 20 °C. Prf fluorescence was excited at 463 nm and measured after passing through a KV500 long pass filter (Schott). Experiments were performed by mixing equal volumes of IC (0.9 µM) with TC-Lys or TC-Phe (0.3 µM) as indicated and monitoring the time course of fluorescence change. Relative fluorescence was calculated by division of all fluorescence values by the value at time 0.

To monitor GTP hydrolysis, peptide bond formation, and mRNA–tRNA translocation following peptide bond formation, equal volumes of IC (0.9 µM) and respective TC (0.3 µM) were mixed in a quench-flow apparatus for time ranging from milliseconds to seconds in TAKM_7_ at 37 °C. For GTP hydrolysis, the reaction was quenched with 50% formic acid. Intact [γ-^32^P]GTP and γ-^32^Pi were separated by TLC in 0.5 M KH_2_PO_4_^27^. The TLC plates were analyzed using phosphorimaging in Typhoon FLA9500 (GE Healthcare). For peptide bond formation and translocation, reactions at specific time points were quenched with 0.5 M KOH and the peptides released by alkaline hydrolysis at 37 °C. Peptide samples were neutralized by adding one fifth volume of 100% glacial acetic acid and analyzed by reversed-phase HPLC (LiChroSpher 100 RP-8 HPLC column, Merck) using 0–65% acetonitrile gradient in 0.1% trifluoroacetic acid. The HPLC fractions were quantified by double label radioactivity counting^49^. The data were normalized to an interval from 0 to 1 and exponential fitting of the data was performed with GraphPad Prism.

### smFRET experiments

Ribosomes labeled at protein L11 and Lys-tRNA^Lys^ labeled with Cy5 at the 3-amino-3-carboxypropyl group at uridine 47 were prepared as described^32,35^. IC was prepared by incubating 70S(L11-Cy3) with a 3-fold excess of IFs, mRNA and f[^3^H]Met-tRNA^fMet^ and GTP (1 mM) for 30 min at 37 °C and purified by centrifugation through sucrose cushion (1.1 M) in TAKM_21_ (50 mM Tris-HCl pH 7.5, 70 mM NH_4_Cl, 30 mM KCl, 21 mM MgCl_2_). The pellet was dissolved in TAKM_7_. TC was prepared by incubating 3-fold excess EF-Tu (or EF-Tu(H84A) mutant) with GTP (1 mM), phosphoenolpyruvate (3 mM), and pyruvate kinase (0.5%) for 15 min at 37 °C and subsequent addition of Lys-tRNA^Lys^(Cy5).

Biotin-coated glass objective slides and coverslips were prepared as described^35^. Reaction chambers were incubated with TAKM_7_ containing putrescine (8 mM), spermidine (1 mM), BSA (10 mg/ml) and neutravidin (1 μM; Thermo Scientific) for 5 min at room temperature. Neutravidin was washed by adding the same buffer containing putrescine (8 mM), spermidine (1 mM) and BSA (1 mg/ml). Purified IC(L11-Cy3) were diluted to 1 nM in TAKM_7_ containing putrescine (8 mM), spermidine (1 mM) and added to the reaction chambers for 2 min at room temperature. Imaging started after addition of TAKM_7_ containing putrescine (8 mM), spermidine (1 mM), protocatechuic acid (2.5 mM), 50 nM *Pseudomonas* protocatechuate-3,4-dioxygenase (50 nM), 6-hydroxy-2,5,7,8-tetramethylchromane-2-carboxylic acid (1 mM) methylviologen (1 mM; Sigma-Aldrich), GTP (1 mM) and TC(Lys-tRNA^Lys^(Cy5)) (5 nM).

TIRF imaging was performed at 22 °C on an IX 81 inverted microscope using a PLAPON 60 × 1.45 numerical aperture objective (Olympus). Cy3 was excited using a 561 nm solid-state laser operated at 25 mW and images were recorded with an electron multiplying CCD (charge-coupled device) camera (CCD-C9100-13, Hamamatsu) at a rate of 30.3 frames/s. Color channels were separated by projecting donor and acceptor emission on different parts of the CCD chip using an image splitter (dual view micro imager DV2, Photometrics), filter specifications HQ 605/40, HQ 680/30 (Chroma Technology).

Fluorescence time courses for Cy3 and Cy5 were extracted using custom-made MATLAB (MathWorks) software according to published protocols^32,35^. A semi-automated algorithm (MATLAB) was used to select single fluorophores showing anticorrelated fluorescence intensities and single-step photobleaching. Cy3 bleed-through into the Cy5 channel was corrected using an experimentally determined coefficient of 0.13. FRET efficiency was calculated as the ratio of the measured emission fluorescence intensities, FI_Cy5_/(FI_Cy3_ + FI_Cy5_). Trajectories were truncated to remove photobleaching and photoblinking events. The set of all FRET traces for a given complex was compiled in a histogram, which was fitted to a sum of Gaussian functions. MATLAB code using an unconstrained nonlinear minimization procedure (fminsearch, MATLAB, R2011b) yields mean values and standard deviation for the distribution of FRET states. Two-dimensional contour plots were generated from raw time-resolved FRET trajectories using a custom-made software. smFRET trajectories were fitted by Hidden Markov model using the vbFRET software package (http://vbfret.sourceforge.net/)^50^ to generate the idealized trajectories. FRET changes in idealized trajectories that were smaller than the s.d. of the Gaussian distribution of the FRET states were not considered transitions because they could not be not distinguished from the noise. Dwell times of different FRET states were calculated from idealized trajectories. The dwell-time distribution was fitted to an exponential function, *y* = *y*_0_ + *Ae*^−*t*/*τ*^ to calculate the decay rate (*k* = 1/*τ*). GraphPad prism 8 software was used for the representation of smFRET data and fits of the data.

### Cryo-EM grid preparation

IC was prepared and purified as described above except that non-radioactive fMet-tRNA^fMet^ was used. TC was prepared from EF-Tu (1.5 µM), EF-Ts (0.02 mM), Lys-tRNA^Lys^ (0.3 µM), GTP (1 mM), phosphoenolpyruvate (3 mM), pyruvate kinase (1%), DTT (1 mM) in buffer TAKM_7_. IC and TC were mixed in a 1:1 molar ratio, diluted to the final concentration of approximately *A*_260_~20 (absorbance at 260 nm 10 mm path) and *A*_280_~10 (absorbance at 280 nm 10 mm path), and incubated on ice for 60 s. The “unreacted” IC complexes contained all the above mentioned components except for Lys-tRNA^Lys^. Approximately 3 μL of the solution was applied onto freshly glow-discharged TEM grids (Quantifoil R2/1, Cu 200 mesh) and plunge-frozen into liquid ethane by a Vitrobot Mark IV (Thermo Fisher Scientific) using the following parameters: humidity 100%, temperature 4 °C, blot total 1,wait time 0, blot force 0, blot time 2 s, drain time 0 s.

### Cryo-EM single-particle reconstruction

Cryo-EM datasets were collected at National Cryo-EM Centre SOLARIS (Kraków, Poland). The datasets of IC + TC complexes with AAA, m^6^AAA, Am^6^AA, and AAm^6^A codons in the A site, as well as AAA unreacted-IC and AAm^6^A unreacted-IC contained 8435, 9366, 7172, 7207, 10115, and 10276 movies, respectively (40 frames each). The movies were acquired using Titan Krios G3i microscope (Thermo Fisher Scientific) operated at 300 kV accelerating voltage, magnification of 105k, and corresponding pixel size of 0.86 Å/px. A K3 direct electron detector used for data collection was fitted with BioQuantum Imaging Filter (Gatan) using 20 eV slit. The K3 detector was operated in a counting mode. Imaged areas were exposed to 40 e^−^/Å^2^ total dose (corresponding to ~16 e^−^/px/s dose rate measured in vacuum). The frame stacks were obtained using under-focus optical conditions with a defocus range of −2.1 to −0.9 µm and 0.3 µm steps. The collected datasets were analyzed with cryoSPARC v3.3.0^51^. Firstly, patch motion correction and patch CTF estimation were performed. Next, approximately 500 particles were picked manually. The acquired sets of particles were subjected to 2D classification and used in the generation of preliminary classes for template picking. The application of a template picker and 2D classification of the datasets resulted in correspondingly 689123, 1113602, 732439, 774481, 1413299, and 1282719 particles, respectively (Supplementary Figs. 3 and 9). These sets were used for Heterogenous Refinement and particles from the classes corresponding to well-defined 70 S ribosomes served as an input for focused 3D classifications. The 3D Classification was performed in cryoSPARC using soft masks corresponding to E-, P- and A-site tRNA generated in Relion 3.1^52^. The unreacted 70S IC particles did not have any discernible density in the E and A sites, as expected for the IC. In the absence of Lys-tRNA^Lys^, the fraction of particles with ordered P-site fMet-tRNA^fMet^ reached 78% (AAA) and 80% (AAm^6^A) in the two datasets, demonstrating the comparable quality of IC preparations. The final particle stacks were unbinned for local motion correction^53^ in cryoSPARC. During final 3D Homogenous Refinements, the particles were subjected to Defocus Refinement, Global CTF Refinement, and Ewald Sphere Correction to generate high-resolution maps. Local map resolution was calculated using cryoSPARC. Prior to model fitting, the combined half-maps were sharpened with DeepEMhancer^54^.

### Molecular modeling

The initial atomic model was assembled by combining core elements of *E. coli* 70S ribosome structures solved by cryo-EM at 2.0 Å (PDB: 7K00^55^) and 2.54 Å resolution (PDB: 6XZB^56^). The initial model of tRNA^fMet^ was sourced from a 3.2 Å ribosome cryo-EM structure (PDB: 6WDD^24^) and tRNA^Lys^ was isolated from a 3.6 Å ribosome cryo-EM reconstruction (PDB: 5JTE^57^). Other structural elements, such as mRNA, were built manually into the map using Coot^58^. Following rigid body fitting using ChimeraX^59^, the atomic coordinates were flexibly fit with Namdinator^60^ and Isolde^61^. The models were real-space refined in Phenix^62^. The final coordinates were validated using MolProbity^63^ and the model statistics are presented in Supplementary Table 1. The cryo-EM maps and atomic models were displayed using ChimeraX version 1.2.5.

### Reporting summary

Further information on research design is available in the Nature Portfolio Reporting Summary linked to this article.

### Supplementary information

Supplementary Information

Peer Review File

Reporting Summary

### Source data

Source Data

## Data Availability

The micrographs, cryo-EM densities, and atomic models generated in this study have been deposited in the Electron Microscopy Public Image Archive (EMPIAR), the Electron Microscopy Data Bank (EMDB), and the Protein Data Bank (PDB) under the following accession codes: (1) AAA EMPIAR-11287; AAA IC EMD-16031 and PDB ID 8BGH; AAA P/P A/A EMD-16015 and PDB ID 8BF7. (2) m^6^AAA EMPIAR-11290; m^6^AAA IC EMD-16065 and PDB ID 8BHP; m^6^AAA P/P A/A EMD-16062 and PDB ID 8BHN. (3) Am^6^AA EMPIAR-11289; Am^6^AA IC EMD 16059 and PDB ID 8BHL; Am^6^AA P/P A/A EMD-16057 and PDB ID 8BHJ. (4) AAm^6^A EMPIAR-11288; AAm^6^A IC EMD-16029 and PDB ID 8BGE; AAm^6^A P/P A/A EMD-16047 and PDB ID 8BH4. (5) AAA unreacted-IC EMPIAR-11291, EMD-16081 and PDB ID 8BIL. (6) AAm^6^A unreacted-IC EMPIAR-11292, EMD-16082 and PDB ID 8BIM. Ensemble kinetics data are provided in the Source Data file. Processed smFRET data are provided in the Source Data file. Original images of smFRET experiments are available upon request due to their large size and the lack of a relevant public database. Source data are provided with this paper.

## Code availability

The codes used to analyze data in this study are available from the corresponding authors upon request.

## References

[1] Wang X (2014). N6-methyladenosine-dependent regulation of messenger RNA stability. Nature.

[2] Lesbirel S (2018). The m(6)A-methylase complex recruits TREX and regulates mRNA export. Sci. Rep..

[3] Uzonyi, A. et al. Exclusion of m6A from splice-site proximal regions by the exon junction complex dictates m6A topologies and mRNA stability. *Mol. Cell* **83**, 237–251 (2022). 10.1016/j.molcel.2022.12.026 36599352

[4] Adhikari S, Xiao W, Zhao YL, Yang YG (2016). m(6)A: Signaling for mRNA splicing. RNA Biol..

[5] Deng X (2015). Widespread occurrence of N6-methyladenosine in bacterial mRNA. Nucleic Acids Res..

[6] Meyer KD (2012). Comprehensive analysis of mRNA methylation reveals enrichment in 3’ UTRs and near stop codons. Cell.

[7] Dominissini D (2012). Topology of the human and mouse m6A RNA methylomes revealed by m6A-seq. Nature.

[8] Wang K, Peng J, Yi C (2021). The m(6)A consensus motif provides a paradigm of epitranscriptomic studies. Biochemistry.

[9] Wei CM, Moss B (1977). Nucleotide sequences at the N6-methyladenosine sites of HeLa cell messenger ribonucleic acid. Biochemistry.

[10] Su S (2022). Cryo-EM structures of human m(6)A writer complexes. Cell Res..

[11] Patil DP, Pickering BF, Jaffrey SR (2018). Reading m(6)A in the transcriptome: m(6)A-binding proteins. Trends Cell Biol..

[12] Zhen D (2020). m(6)A Reader: epitranscriptome target prediction and functional characterization of N (6)-methyladenosine (m(6)A) readers. Front. Cell Dev. Biol..

[13] Yen YP, Chen JA (2021). The m(6)A epitranscriptome on neural development and degeneration. J. Biomed. Sci..

[14] Liu S (2021). Role of RNA N6-methyladenosine modification in male infertility and genital system tumors. Front. Cell Dev. Biol..

[15] Wang T, Kong S, Tao M, Ju S (2020). The potential role of RNA N6-methyladenosine in Cancer progression. Mol. Cancer.

[16] Mao Y (2019). m(6)A in mRNA coding regions promotes translation via the RNA helicase-containing YTHDC2. Nat. Commun..

[17] Rodnina, M. V. Translation in prokaryotes. *Cold Spring Harb. Perspect. Biol.* **10**, 1–21 (2018). 10.1101/cshperspect.a032664 PMC6120702 29661790

[18] Rodnina MV (2023). Decoding and recoding of mRNA sequences by the ribosome. Annu. Rev. Biophys..

[19] Hoernes TP (2016). Nucleotide modifications within bacterial messenger RNAs regulate their translation and are able to rewire the genetic code. Nucleic Acids Res..

[20] Choi J (2016). N(6)-methyladenosine in mRNA disrupts tRNA selection and translation-elongation dynamics. Nat. Struct. Mol. Biol..

[21] Ieong KW, Indrisiunaite G, Prabhakar A, Puglisi JD, Ehrenberg M (2021). N 6-Methyladenosines in mRNAs reduce the accuracy of codon reading by transfer RNAs and peptide release factors. Nucleic Acids Res..

[22] Ranjan N, Rodnina MV (2017). Thio-Modification of tRNA at the Wobble Position as Regulator of the Kinetics of Decoding and Translocation on the Ribosome. J. Am. Chem. Soc..

[23] Rodnina, M. V., Fischer, N., Maracci, C. & Stark, H. Ribosome dynamics during decoding. *Philos. Trans. R. Soc. Lond. B Biol. Sci.* **372**, 1–10 (2017). 10.1098/rstb.2016.0182 PMC5311926 28138068

[24] Loveland AB, Demo G, Korostelev AA (2020). Cryo-EM of elongating ribosome with EF-Tu*GTP elucidates tRNA proofreading. Nature.

[25] Fischer N (2016). The pathway to GTPase activation of elongation factor SelB on the ribosome. Nature.

[26] Kothe U, Rodnina MV (2006). Delayed release of inorganic phosphate from elongation factor Tu following GTP hydrolysis on the ribosome. Biochemistry.

[27] Gromadski KB, Rodnina MV (2004). Kinetic determinants of high-fidelity tRNA discrimination on the ribosome. Mol. Cell.

[28] Rodnina MV, Pape T, Fricke R, Kuhn L, Wintermeyer W (1996). Initial binding of the elongation factor Tu.GTP.aminoacyl-tRNA complex preceding codon recognition on the ribosome. J. Biol. Chem..

[29] Huang B, Johansson MJ, Bystrom AS (2005). An early step in wobble uridine tRNA modification requires the Elongator complex. RNA.

[30] Leidel S (2009). Ubiquitin-related modifier Urm1 acts as a sulphur carrier in thiolation of eukaryotic transfer RNA. Nature.

[31] Rezgui VA (2013). tRNA tKUUU, tQUUG, and tEUUC wobble position modifications fine-tune protein translation by promoting ribosome A-site binding. Proc. Natl Acad. Sci. USA.

[32] Adio S (2015). Fluctuations between multiple EF-G-induced chimeric tRNA states during translocation on the ribosome. Nat. Commun..

[33] Geggier P (2010). Conformational sampling of aminoacyl-tRNA during selection on the bacterial ribosome. J. Mol. Biol..

[34] Chen C (2011). Single-molecule fluorescence measurements of ribosomal translocation dynamics. Mol. Cell.

[35] Poulis P, Patel A, Rodnina MV, Adio S (2022). Altered tRNA dynamics during translocation on slippery mRNA as determinant of spontaneous ribosome frameshifting. Nat. Commun..

[36] Daviter T, Wieden HJ, Rodnina MV (2003). Essential role of histidine 84 in elongation factor Tu for the chemical step of GTP hydrolysis on the ribosome. J. Mol. Biol..

[37] Ogle JM (2001). Recognition of cognate transfer RNA by the 30 S ribosomal subunit. Science.

[38] Demeshkina N, Jenner L, Westhof E, Yusupov M, Yusupova G (2012). A new understanding of the decoding principle on the ribosome. Nature.

[39] Chandrasekaran V (2019). Mechanism of ribosome stalling during translation of a poly(A) tail. Nat. Struct. Mol. Biol..

[40] Tesina P (2020). Molecular mechanism of translational stalling by inhibitory codon combinations and poly(A) tracts. EMBO J..

[41] Roost C (2015). Structure and thermodynamics of N6-methyladenosine in RNA: a spring-loaded base modification. J. Am. Chem. Soc..

[42] Rozov A (2018). Tautomeric G*U pairs within the molecular ribosomal grip and fidelity of decoding in bacteria. Nucleic Acids Res..

[43] Loveland AB, Demo G, Grigorieff N, Korostelev AA (2017). Ensemble cryo-EM elucidates the mechanism of translation fidelity. Nature.

[44] Ogle JM, Murphy FV, Tarry MJ, Ramakrishnan V (2002). Selection of tRNA by the ribosome requires a transition from an open to a closed form. Cell.

[45] Ogle JM, Ramakrishnan V (2005). Structural insights into translational fidelity. Annu. Rev. Biochem..

[46] Gromadski KB, Daviter T, Rodnina MV (2006). A uniform response to mismatches in codon-anticodon complexes ensures ribosomal fidelity. Mol. Cell.

[47] Rodnina MV, Fricke R, Kuhn L, Wintermeyer W (1995). Codon-dependent conformational change of elongation factor Tu preceding GTP hydrolysis on the ribosome. EMBO J..

[48] Wintermeyer W, Zachau HG (1979). Fluorescent derivatives of yeast tRNAPhe. Eur. J. Biochem..

[49] Wohlgemuth I, Brenner S, Beringer M, Rodnina MV (2008). Modulation of the rate of peptidyl transfer on the ribosome by the nature of substrates. J. Biol. Chem..

[50] Bronson JE, Fei J, Hofman JM, Gonzalez RL, Wiggins CH (2009). Learning rates and states from biophysical time series: a Bayesian approach to model selection and single-molecule FRET data. Biophys. J..

[51] Punjani A, Rubinstein JL, Fleet DJ, Brubaker MA (2017). cryoSPARC: algorithms for rapid unsupervised cryo-EM structure determination. Nat. Methods.

[52] Zivanov J, Nakane T, Scheres SHW (2020). Estimation of high-order aberrations and anisotropic magnification from cryo-EM data sets in RELION-3.1. IUCrJ.

[53] Rubinstein JL, Brubaker MA (2015). Alignment of cryo-EM movies of individual particles by optimization of image translations. J. Struct. Biol..

[54] Sanchez-Garcia R (2021). DeepEMhancer: a deep learning solution for cryo-EM volume post-processing. Commun. Biol..

[55] Watson, Z. L. et al. Structure of the bacterial ribosome at 2A resolution. *Elife* **9**, e60482 (2020). 10.7554/eLife.60482 PMC7550191 32924932

[56] Pichkur EB (2020). Insights into the improved macrolide inhibitory activity from the high-resolution cryo-EM structure of dirithromycin bound to the E. coli 70S ribosome. RNA.

[57] Arenz S (2016). A combined cryo-EM and molecular dynamics approach reveals the mechanism of ErmBL-mediated translation arrest. Nat. Commun..

[58] Emsley P, Lohkamp B, Scott WG, Cowtan K (2010). Features and development of Coot. Acta Crystallogr. D. Biol. Crystallogr..

[59] Pettersen EF (2021). UCSF ChimeraX: structure visualization for researchers, educators, and developers. Protein Sci..

[60] Kidmose RT (2019). Namdinator - automatic molecular dynamics flexible fitting of structural models into cryo-EM and crystallography experimental maps. IUCrJ.

[61] Croll TI (2018). ISOLDE: a physically realistic environment for model building into low-resolution electron-density maps. Acta Crystallogr. D Struct. Biol..

[62] Adams PD (2010). PHENIX: a comprehensive Python-based system for macromolecular structure solution. Acta Crystallogr. D. Biol. Crystallogr..

[63] Davis IW (2007). MolProbity: all-atom contacts and structure validation for proteins and nucleic acids. Nucleic Acids Res..

[64] Petrychenko V (2021). Structural mechanism of GTPase-powered ribosome-tRNA movement. Nat. Commun..

[65] Carbone CE (2021). Time-resolved cryo-EM visualizes ribosomal translocation with EF-G and GTP. Nat. Commun..

[66] Rundlet EJ (2021). Structural basis of early translocation events on the ribosome. Nature.

