# Supplementary material for: Modulation of translational decoding by m6A modification of mRNA
Source: Nat Commun. 2023 Aug 8;14:4784. doi: 10.1038/s41467-023-40422-7 (PMC10409866; doi:10.1038/s41467-023-40422-7)
Supplement: Supplementary file 1 — Supplementary Information [file 41467_2023_40422_MOESM1_ESM.pdf]

Supplementary Information for

Modulation of translational decoding by m^{6}A modification of mRNA

Sakshi Jain[1,*], Lukasz Koziej[2,*], Panagiotis Poulis[1,*], Igor Kaczmarczyk[2,3], Monika Gaik[2], Michał Rawski[2,4], Namit Ranjan[1], Sebastian Glatt[2,#] and Marina V. Rodnina[1,#]

[1] Max Planck Institute for Multidisciplinary Sciences, Göttingen, 37077, Germany

[2] Malopolska Centre of Biotechnology, Jagiellonian University, Krakow, 30-387, Poland

[3] Doctoral School of Exact and Natural Sciences, Jagiellonian University, Krakow, 30-387, Poland

[4] National Synchrotron Radiation Centre SOLARIS, Jagiellonian University, Krakow, 30-387, Poland

Supplementary Table 1. Cryo-EM data collection and structure refinement statistics.

	AAA		m6AAA		Am6AA		AAm6A		AAA	AAm6A
	IC	P/P A/A	IC	P/P A/A	IC	P/P A/A	IC	P/P A/A	Unreacted IC	Unreacted IC
**EMDB**	16031	16015	16065	16062	16059	16057	16029	16047	16081	16082
**PDB**	8BGH	8BF7	8BHP	8BHN	8BHL	8BHJ	8BGE	8BH4	8BIL	8BIM
**Data collection and processing**										
Magnification	105,000 ×	105,000 ×	105,000 ×	105,000 ×	105,000 ×	105,000 ×	105,000 ×	105,000 ×	105,000 ×	105,000 ×
Voltage (keV)	300	300	300	300	300	300	300	300	300	300
Electron exposure (e-/Å^2)	40	40	40	40	40	40	40	40	40	40
Defocus range	-0.9 to -2.1	-0.9 to -2.1	-0.9 to -2.1	-0.9 to -2.1	-0.9 to -2.1	-0.9 to -2.1	-0.9 to -2.1	-0.9 to -2.1	-0.9 to -2.1	-0.9 to -2.1
Pixel size (Å)	0.86	0.86	0.86	0.86	0.86	0.86	0.86	0.86	0.86	0.86
Symmetry imposed	C1	C1	C1	C1	C1	C1	C1	C1	C1	C1
Initial particle images (no.)	689,123	689,123	1,113,602	1,113,602	732,439	732,439	774,481	774,481	1,413,299	1,282,719
Final particle images (no.)	17,151	103,992	334,372	41,219	244,504	24,054	271,987	29,614	778,596	670,987
Map resolution (Å)	2.88	2.33	2.37	2.85	2.21	2.81	2.11	2.62	2.04	2.04
FSC threshold	0.143	0.143	0.143	0.143	0.143	0.143	0.143	0.143	0.143	0.143
Map resolution range (Å)	2.8 – 6.5	2.3 – 4.6	2.3 – 3.5	2.8 – 5.8	2.2 – 3.8	2.8 – 6.3	2.1 – 3.4	2.6 – 5.8	1.9 – 2.9	1.9 – 2.9
Guinier plot Bfactor	26.4	42.2	56.8	40.9	45.6	31.4	43.9	35.6	50.5	47.5
PDB codes of the initial models (extracted molecule): 7K00 (70S), 6XZB (70S),6WDD (tRNA^{fMet}) and 5JTE (tRNA^{Lys})										
Model resolution (Å)	2.88	2.33	2.37	2.85	2.21	2.81	2.11	2.62	2.04	2.04
FSC threshold	0.143	0.143	0.143	0.143	0.143	0.143	0.143	0.143	0.143	0.143
Model composition										
Non-hydrogen atoms	141,020	142,691	141,062	142,692	140,582	142,211	140,582	142,211	141,062	141,063
Protein residues	5682	5683	5682	5683	5622	5623	5622	5623	5682	5682
Nucleotides	4497	4575	4499	4575	4499	4575	4499	4575	4499	4499
B factors (Å^2)										
Protein	53.42	48.03	50.05	58.74	42.54	65.72	39.84	60.44	36.67	36.47
Nucleotide	50.41	46.82	47.29	54.57	39.97	56.78	37.74	55.08	34.49	33.54
R.M.S. deviations										
Bond lengths (Å)	0.005	0.004	0.006	0.003	0.006	0.003	0.007	0.004	0.004	0.005
Bond angles (°)	0.650	0.605	0.682	0.536	0.724	0.553	0.802	0.617	0.683	0.711
Validation										
MolProbity score	1.47	1.36	1.25	1.42	1.24	1.52	1.25	1.52	1.21	1.19
Clashscore	2.74	2.31	1.67	2.96	1.63	3.63	1.66	3.35	1.57	1.46
Poor rotamers (%)	0.02	0.75	0.62	0.04	0.57	0.00	0.44	0.02	0.47	0.45
Ramachandran										
Favored (%)	93.94	94.91	95.18	95.14	95.35	94.61	95.22	94.08	95.50	95.54
Allowed (%)	6.06	5.09	4.82	4.84	4.65	5.39	4.78	5.92	4.50	4.46
Disallowed (%)	0.00	0.00	0.00	0.02	0.00	0.00	0.00	0.00	0.00	0.00
CC volume	0.84	0.85	0.87	0.84	0.86	0.83	0.87	0.84	0.86	0.86

Supplementary Figures

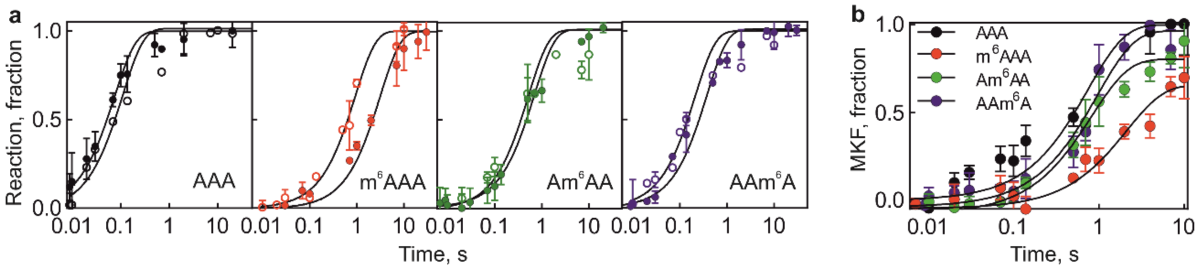

Supplementary Fig. 1. m^{6}A modification delays mRNA decoding and mRNA–tRNA translocation. a, GTP hydrolysis (open circles) and peptide bond formation (closed circles) measured upon mixing of IC (0.9 µM) with EF-Tu–[γ-^{32}P]GTP–[^{14}C]Lys-tRNA^{Lys} or EF-Tu–GTP–[^{14}C]Lys-tRNA^{Lys} (0.3 µM), respectively. [γ-^{32}P]GTP and γ-^{32}P$_i$ were separated by TLC plates and quantified by phosphorimaging using Typhoon FLA9500. f[^{3}H]Met-[^{14}C]Lys dipeptides were separated from amino acids by HPLC and quantified using scintillation counting. The k_{GTP} and k_{pep} (s^{-1}) from exponential fitting are 9 ± 2 (AAA), 1 ± 0.1 (m^{6}AAA), 1.9 ± 0.5 (Am^{6}AA), 4.7 ± 1 (AAm^{6}A) and 13 ± 1.4 (AAA), 0.3 ± 0.04 (m^{6}AAA), 1.4 ± 0.1 (Am^{6}AA), 2.6 ± 0.3 (AAm^{6}A) respectively. **b,** Time courses of f[^{3}H]Met-[^{14}C]Lys-Phe formation obtained by mixing ribosome complexes with tRNA^{fMet} in the P site and f[^{3}H]Met-[^{14}C]Lys-tRNA^{Lys} in the A site (0.3 µM) with EF-Tu–GTP–[^{14}C]Phe-tRNA^{Phe} (0.25 µM) and EF-G (4 µM). f[^{3}H]Met-[^{14}C]Lys-Phe tripeptides were separated from amino acids and dipeptides by HPLC and quantified by scintillation counting. For **a** and **b**, each time course is the mean of 3 independent experiments with error bars representing the standard deviation (N=3). Smooth black lines represent single exponential fits. Source data are provided as a Source Data file.

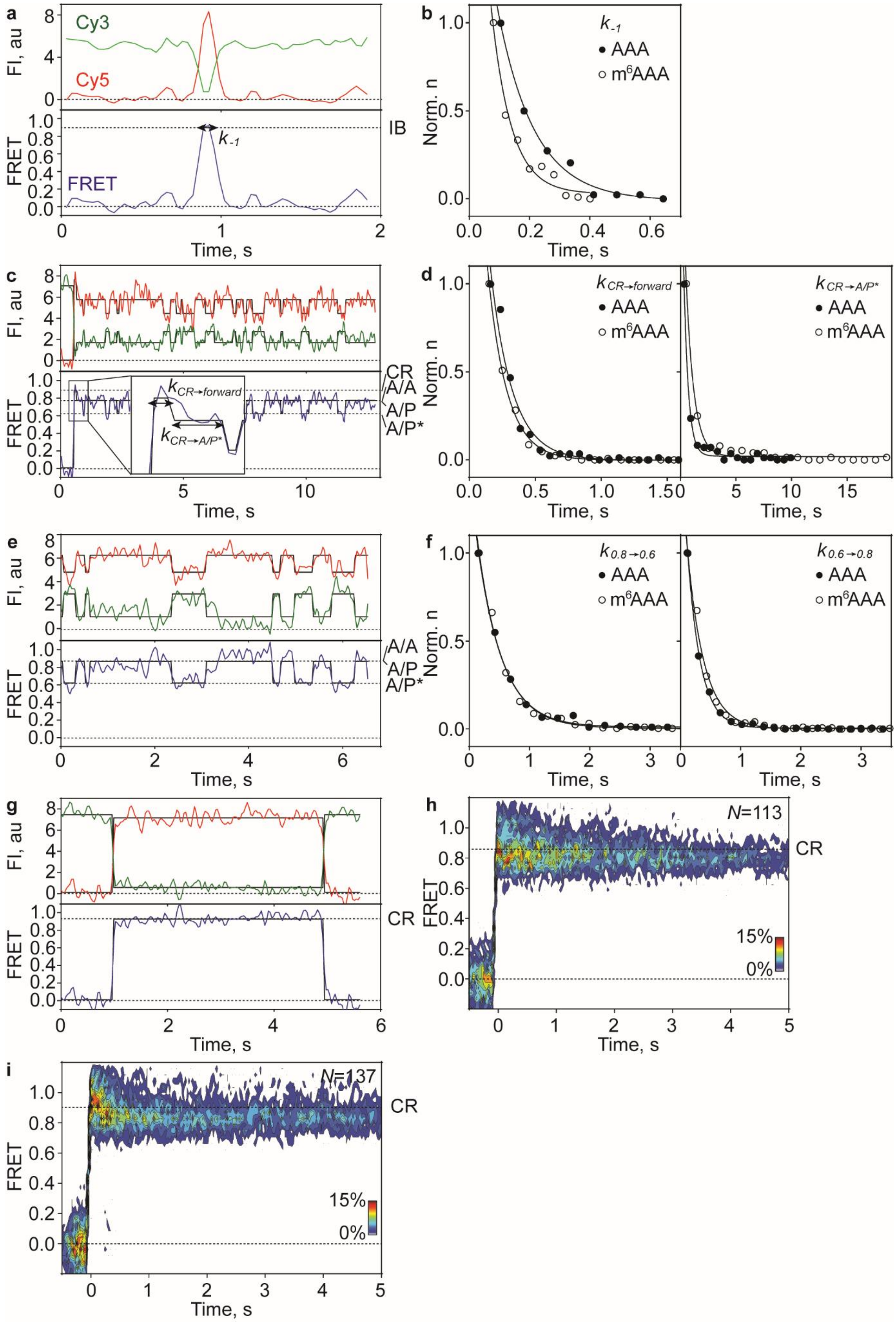
a
Cy3
Cy5
FI, au
FRET
k-1
IB
Time, s
b
Norm. n
k-1
AAA
m6AAA
Time, s
c
CR
A/A
A/P
A/P*
kCR→forward
kCR→A/P*
d
kCR→forward
kCR→A/P*
e
f
k0.8→0.6
k0.6→0.8
g
h
N=113
15%
0%
i
N=137

◀**Supplementary Fig. 2. Kinetic analysis of decoding of AAA and m^{6}AAA. a**, Representative smFRET time trace corresponding to the initial binding (IB) and dissociation (k_{-1}) of EF-Tu(wt)–GTP–Lys-tRNA^{Lys}(Cy5) on the AAA codon. Donor Cy3 (green) and acceptor Cy5 (red) fluorescence intensities (FI; au, arbitrary units), and the calculated FRET (blue) are shown with the same color code throughout. The trace shows progression from FRET 0.0 (IC) to FRET 0.9 (IB) followed by return to FRET 0.0 (dissociation). **b**, Normalized dwell-time histogram of dissociation rate (k_{-1}) of TC during decoding of AAA (closed circles) and m^{6}AAA (open circles) codon (**Table 1**). n, number of transitions. Normalization was performed by division by the maximum value of the histogram. Black lines show single exponential curve fits. **c**, Representative smFRET time trace showing the accommodation of Lys-tRNA^{Lys}(Cy5) on the AAA codon. The trace shows progression from FRET 0.0 (IC) to FRET 0.9 (CR) followed by fluctuations between FRET 0.8 (A/A, A/P) and 0.6 (A/P*) states. Black line indicates Hidden Markov Model fit. Zoom-in shows the part of the trace that corresponds to the accommodation ($k_{CR \rightarrow forward}$) and the transition from post-decoding (FRET 0.8) to A/P* (FRET 0.6) state ($k_{CR \rightarrow A/P*}$). **d**, Normalized dwell-time histograms of the transition from the FRET 0.9 (CR) to the post-decoding (FRET 0.8) state ($k_{CR \rightarrow forward}$) and from the disappearance of the FRET 0.9 state (CR) to FRET 0.6 (A/P*) state ($k_{CR \rightarrow A/P*}$) during decoding of AAA (closed circles) and m^{6}AAA (open circles) codon (**Table 1**). **e**, Representative smFRET time trace showing tRNA fluctuations in the post-decoding complex on the AAA codon between FRET 0.8 (A/A, A/P) and 0.6 (A/P*). **f**, Normalized dwell-time histograms of the transitions from the FRET 0.8 (A/A, A/P) to FRET 0.6 (A/P*) state ($k_{0.8 \rightarrow 0.6}$) and from the FRET 0.6 (A/P*) to FRET 0.8 (A/A, A/P) state ($k_{0.6 \rightarrow 0.8}$) in the PRE complex during decoding of AAA (closed circles) and m^{6}AAA (open circles) codon (**Table 1**). **g**, Representative smFRET time trace corresponding to codon reading (CR) without accommodation of Lys-tRNA^{Lys}(Cy5) on the AAA codon. The trace shows progression from FRET 0.0 (IC) to long-lived FRET 0.9 (CR) state without further progression to FRET 0.8 (A/A, A/P) and 0.6 (A/P*) states. **h**, Contour plot showing the distribution of FRET values after synchronization during CR without progression to Lys-tRNA^{Lys}-Cy5 accommodation in the A site carrying AAA codon. Traces are synchronized to the first transition with FRET>0. Data are from 3 independent experiments. *N*, number of traces. **i**, Contour plot showing the distribution of FRET values after synchronization during CR without progression to accommodation of Lys-tRNA^{Lys}(Cy5) in the A site carrying m^{6}AAA codon. Data are from 3 independent experiments. *N*, number of traces. Source data are provided as a Source Data file.

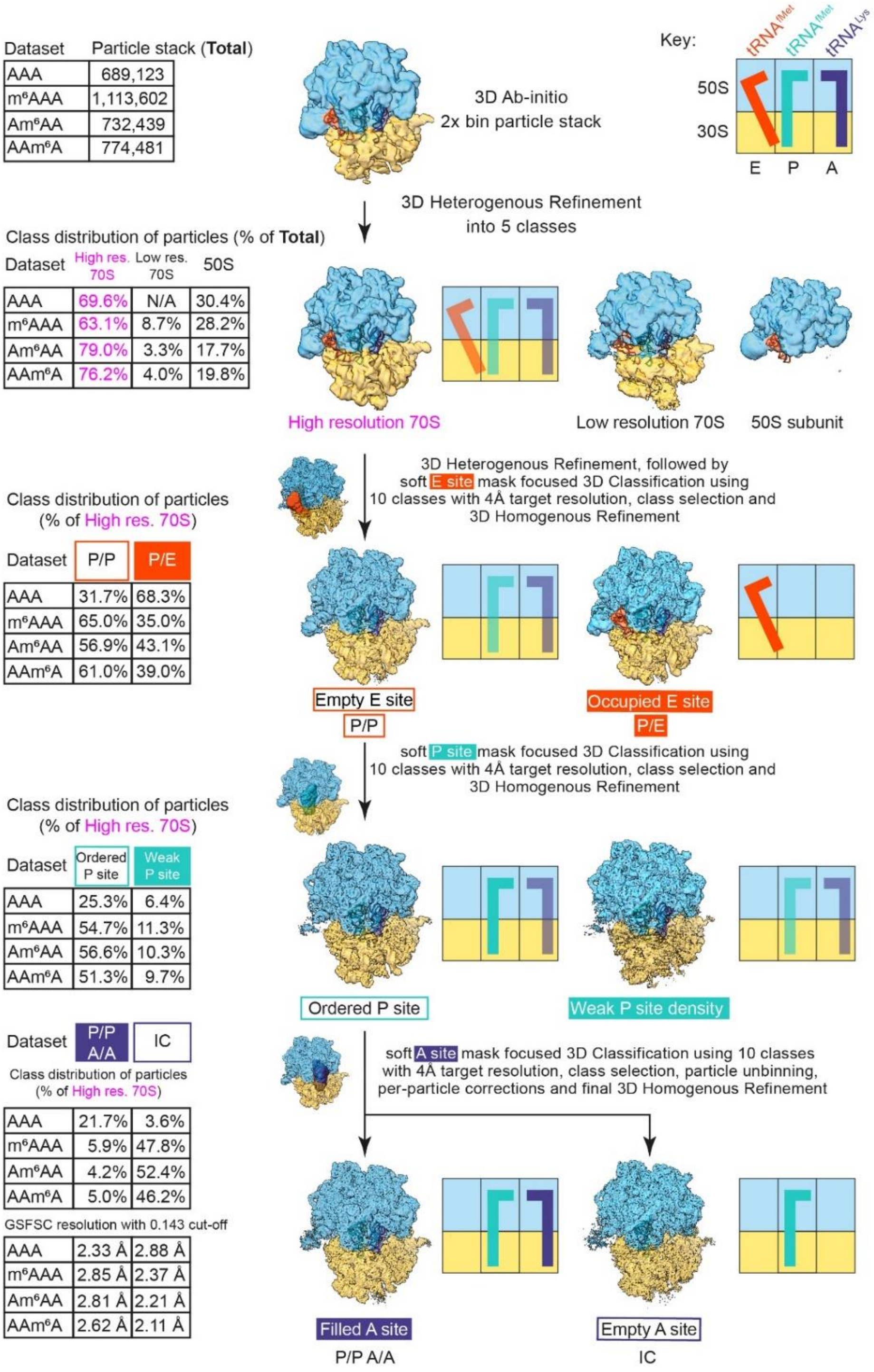

Supplementary Fig. 3. 3D classification procedure of cryo-EM data. The schematic depicts a focused classification strategy applied to resolve conformational heterogeneity of ribosomes and partial occupancy of tRNAs (blurred sticks). In round 1 of the classification, particles displaying hybrid P/E-site tRNA densities within the E-site masked area were eliminated. In round 2, we applied a soft mask in the P site and removed a small fraction of particles with weak $tRNA^{fMet}$ occupancy. In round 3, to ultimately sort out IC that failed to accommodate $tRNA^{Lys}$, a soft mask was used on the A site and particles without a detectable density were excluded. The applied stepwise classification strategy produced ribosome complexes in a non-rotated state with deacylated $tRNA^{fMet}$ in P site and dipeptidyl $tRNA^{Lys}$ in the A site. The examples of structures are from the AAA or AAm^6A datasets.

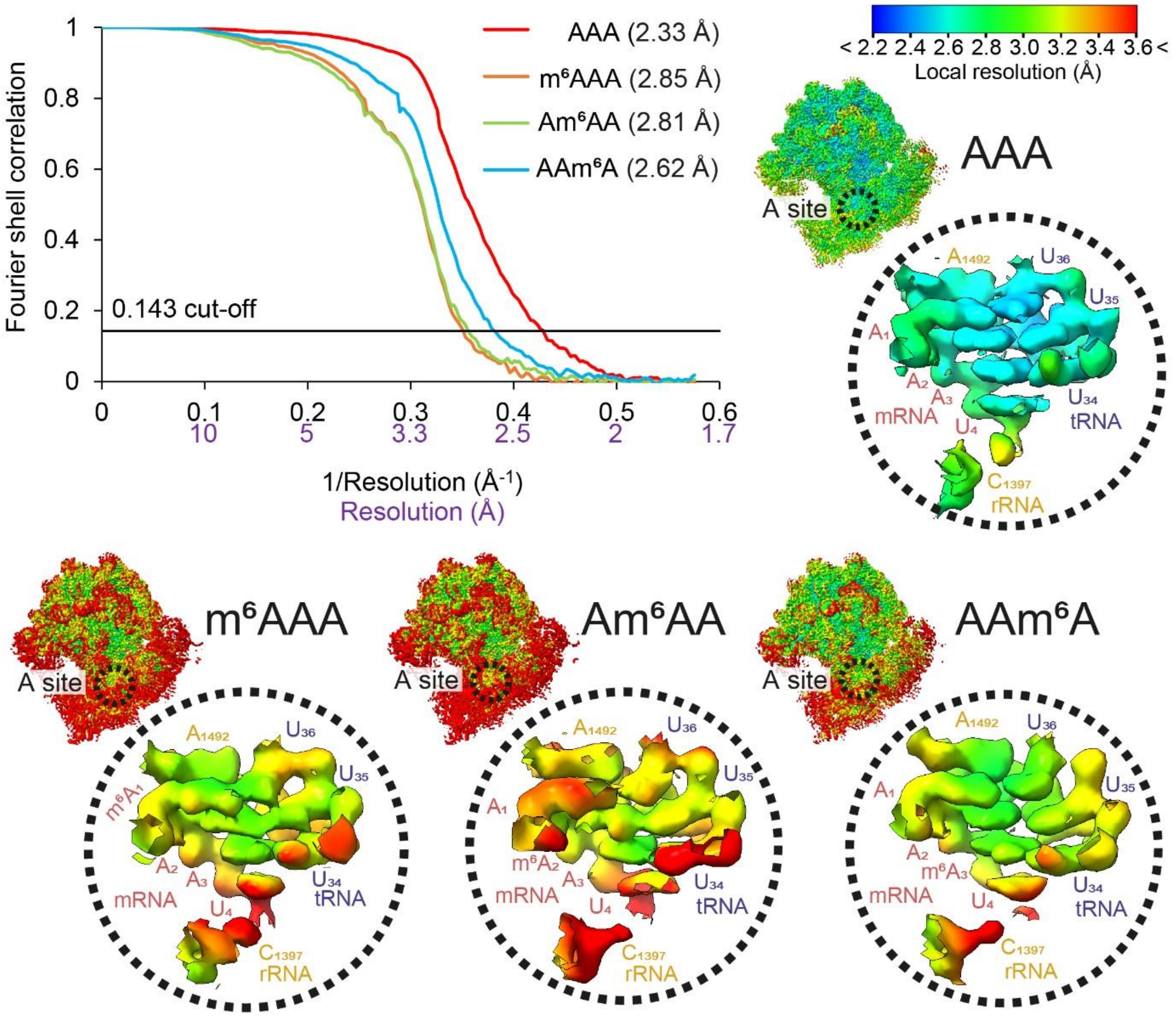

Supplementary Fig. 4. Global and local resolution estimations of cryo-EM maps for P/P A/A state. Fourier shell correlation (FSC) for cryo-EM maps of complexes with unmodified AAA, as well as AAm^{6}A, Am^{6}AA, and m^{6}AAA codons. The global resolution indicated for final maps was estimated using a 0.143 FSC cut-off. Filtered maps illustrate the local resolution near the decoding site (circled and enlarged). The threshold level of the maps was adjusted individually.

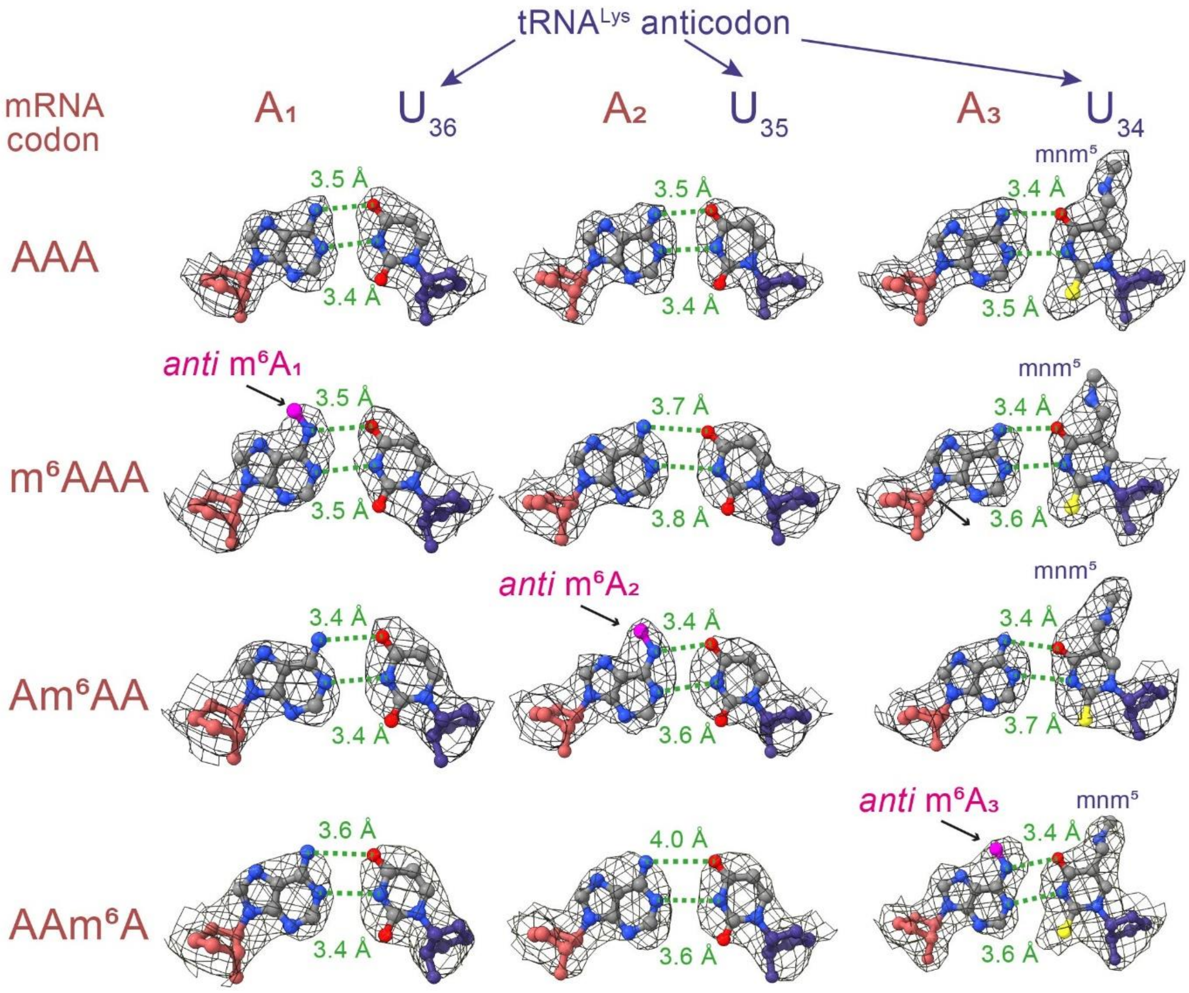

Supplementary Fig. 5. Map quality of post-decoding complexes with fMet-Lys-tRNA^{Lys} in the A site. Close-up view of single nucleotide bases within the codon-anticodon region (each pair was extracted from the structural context and flattened). The measured distances (in Å) between the conventional hydrogen bond donor and acceptor are indicated with green dashed lines. The cryo-EM maps are shown as a mesh using volume threshold rmsLevel4.

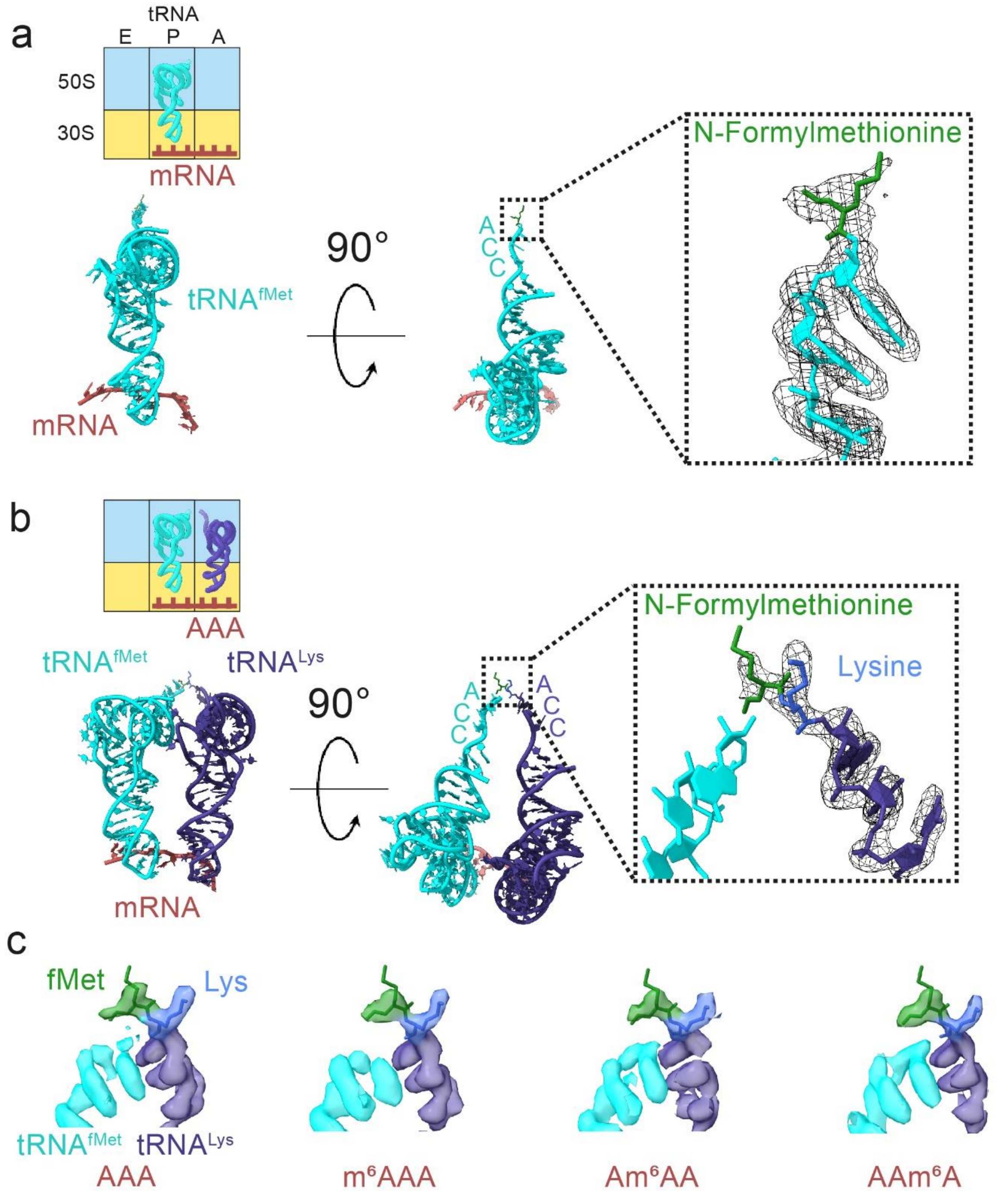

Supplementary Fig. 6. Cryo-EM densities of the fMet-Lys moiety in the peptidyl transferase center of the ribosome. **a,** IC with fMet-tRNA^{fMet} in the P site. **b,** Post-decoding complex with deacylated tRNA^{fMet} in the P site and fMet-Lys-tRNA^{Lys} in the A site. **c,** Comparison of 3′ ends of the A-site fMet-Lys-tRNA^{Lys} between ribosomes with unmodified and m^6-modified AAA codon in different positions. Cryo-EM maps prior to DeepEMhancer sharpening were used to visualize the 3′ ends of tRNA. The densities are displayed using volume threshold rmsLevel 5.

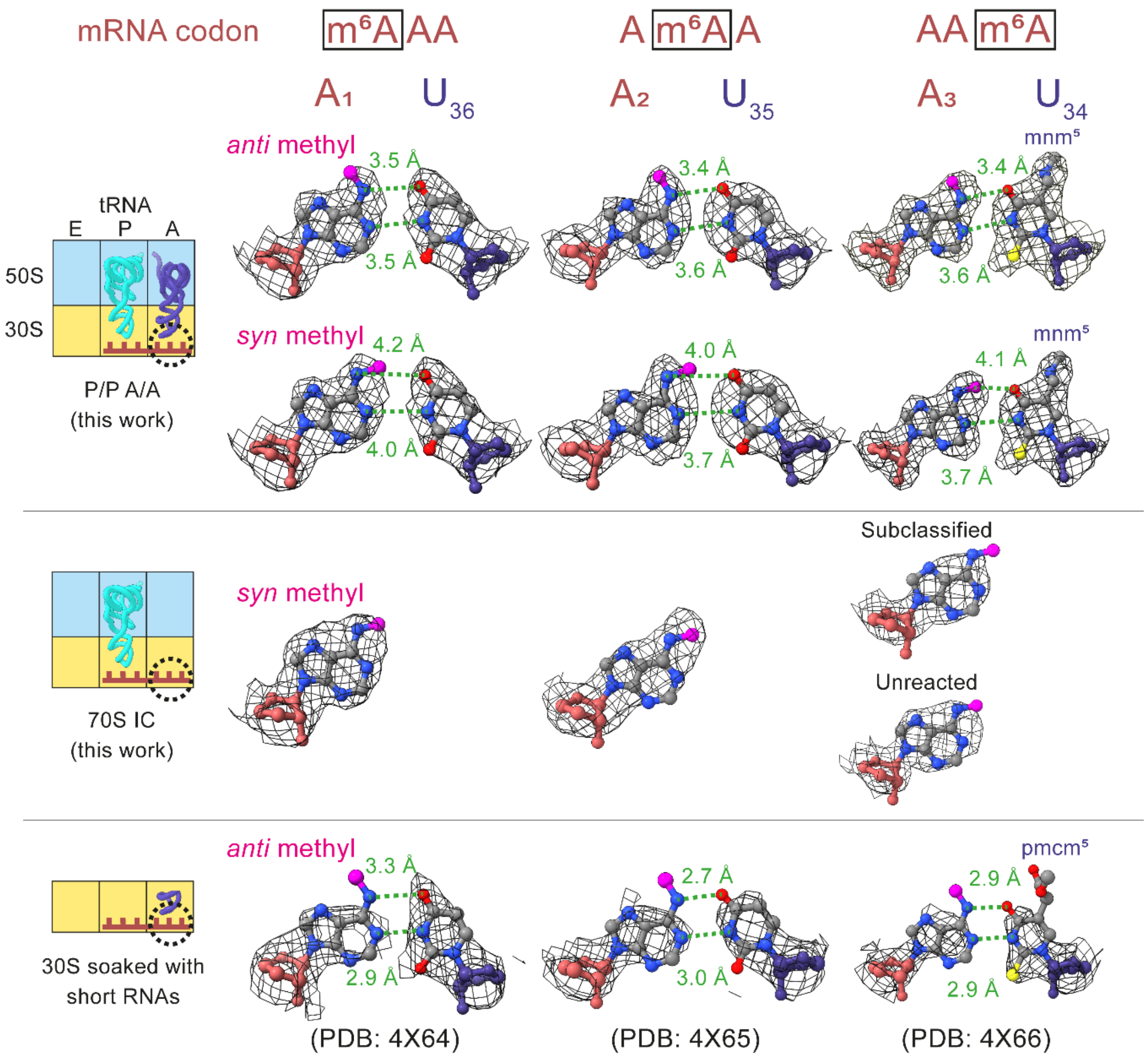

Supplementary Figure 7. Close-up comparison of *syn* and *anti* m^{6}A geometry (m^6 in magenta). Nucleotides containing m^{6}A-modification in the A site (red ribose) were extracted from the structural context and flattened together with the tRNA anticodon (dark blue ribose) or without a base pair (70S IC). The structures of post-decoding ribosome complexes with m^{6}A *anti* and *syn* conformation were independently refined in 3D space resulting in a shift of the entire nucleotides within the available density. The measured distances expressed in Å between the conventional hydrogen bond donors and acceptors are indicated with green dashed lines. The maps are shown as a mesh. Map quality around single nucleotide bases within codon region is shown. The maps of complexes with tRNA^{Lys} in the A site are displayed using threshold rmsLevel 4. The 70S IC maps are displayed using threshold rmsLevel 2. For comparison, electron densities of *T. thermophilus* 30S subunit [18] were displayed using threshold rmsLevel 1.5.

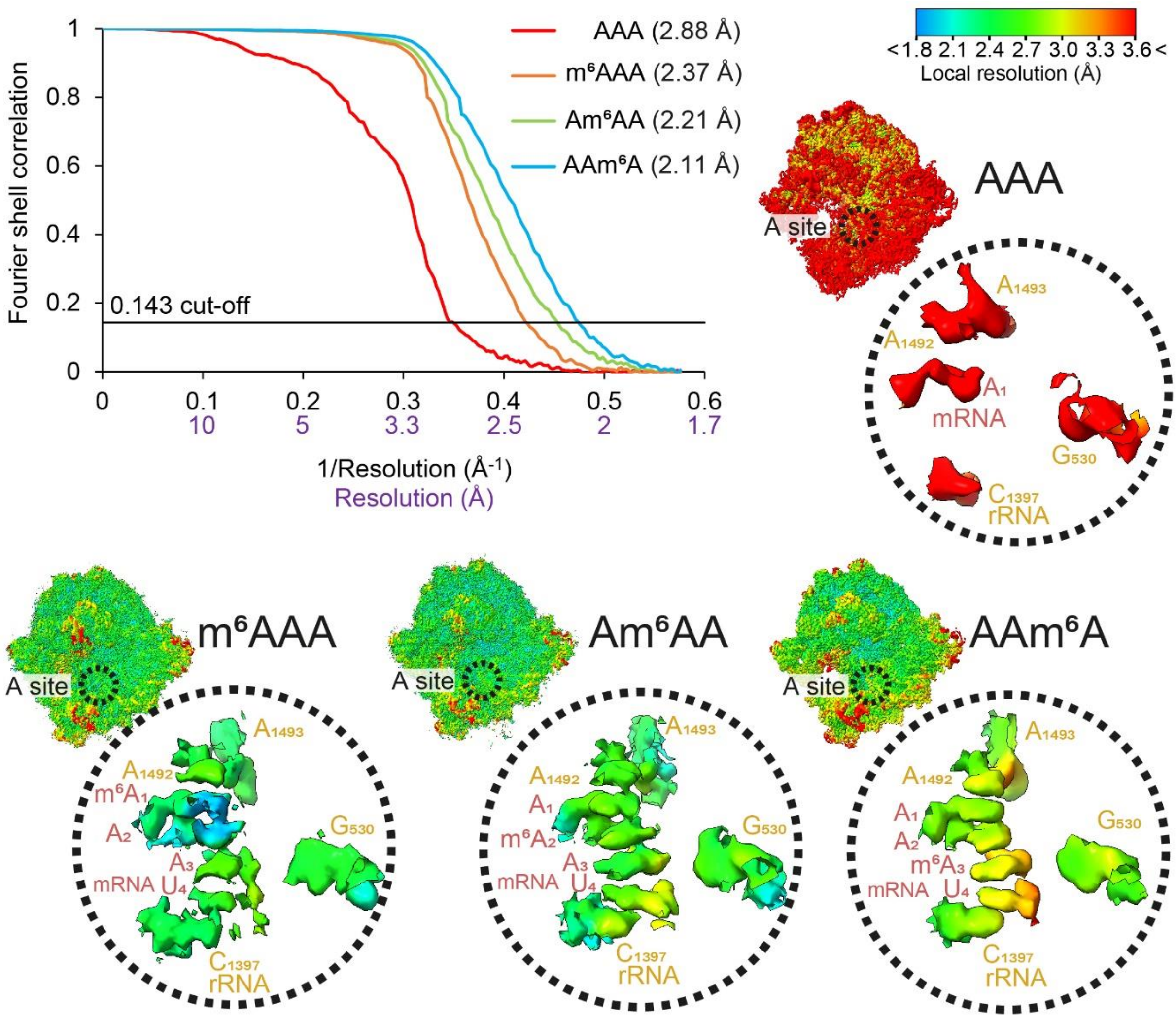

Supplementary Fig. 8. Global and local resolution estimation of cryo-EM maps for subclassified ICs. Fourier shell correlation (FSC) between half maps corresponding to subclassified 70S IC with unmodified AAA, AAm^{6}A, Am^{6}AA, or m^{6}AAA codon and fMet-tRNA^{fMet} in the P site. The global resolution indicated for final maps was estimated using 0.143 FSC cut-off. Filtered maps showing the local resolution near the decoding site (circled and enlarged). The threshold level of the maps was adjusted individually.

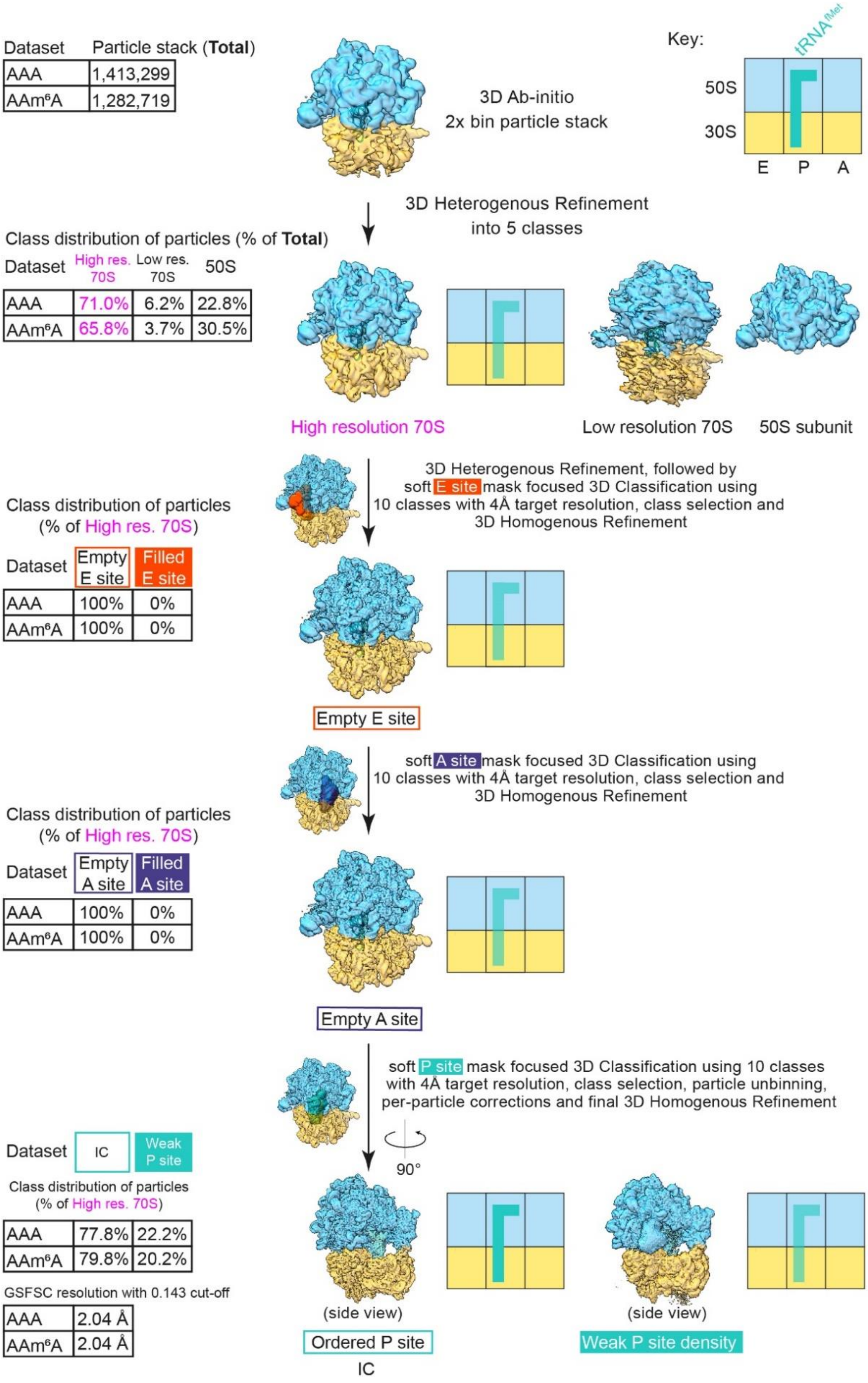

Supplementary Fig. 9. 3D classification procedure of cryo-EM data for unreacted IC. The schematic depicts a focused classification strategy aimed at obtaining high-resolution structures of unreacted IC (Ordered P site) with AAA or AAm^{6}A codon. This general scheme was exemplified by showing selected maps from the unreacted IC AAA dataset.

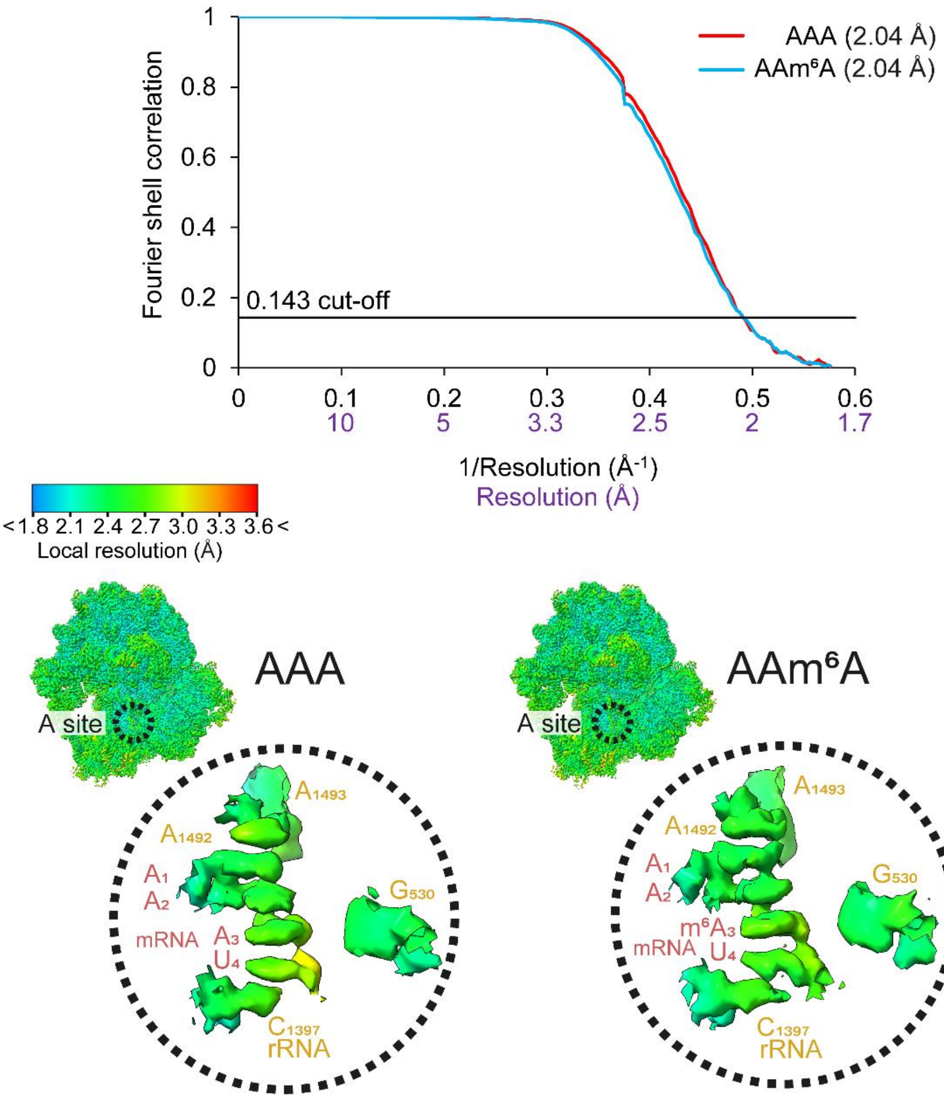

Supplementary Fig. 10. Global and local resolution estimation of cryo-EM maps for unreacted IC. Fourier shell correlation (FSC) between half maps of 70S IC with AAA, or AAm^{6}A in the A site. The global resolution indicated for final maps was estimated using 0.143 FSC cut-off. Filtered maps show the local resolution near the decoding site (circled and enlarged). The threshold level of the maps was adjusted individually.

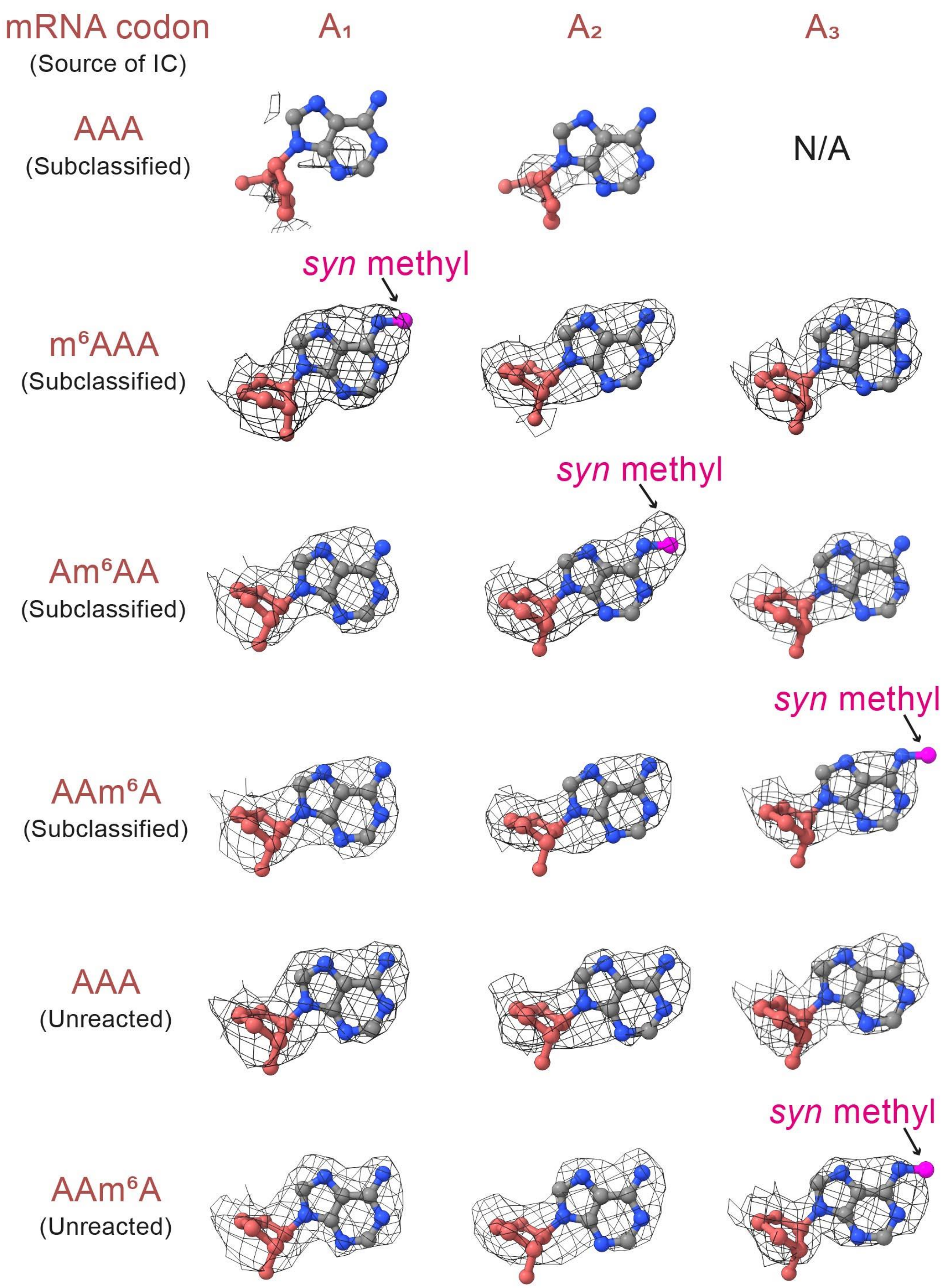

Supplementary Fig. 11. The quality of the cryo-EM map around the A-site codons in various ICs. The available cryo-EM density precludes unambiguous identification of the m^6A modifications (magenta). Close-up view of single nucleotide bases within codon region is shown (each nucleotide was extracted from the structural context and flattened). The maps are shown as mesh using volume threshold rmsLevel 2.

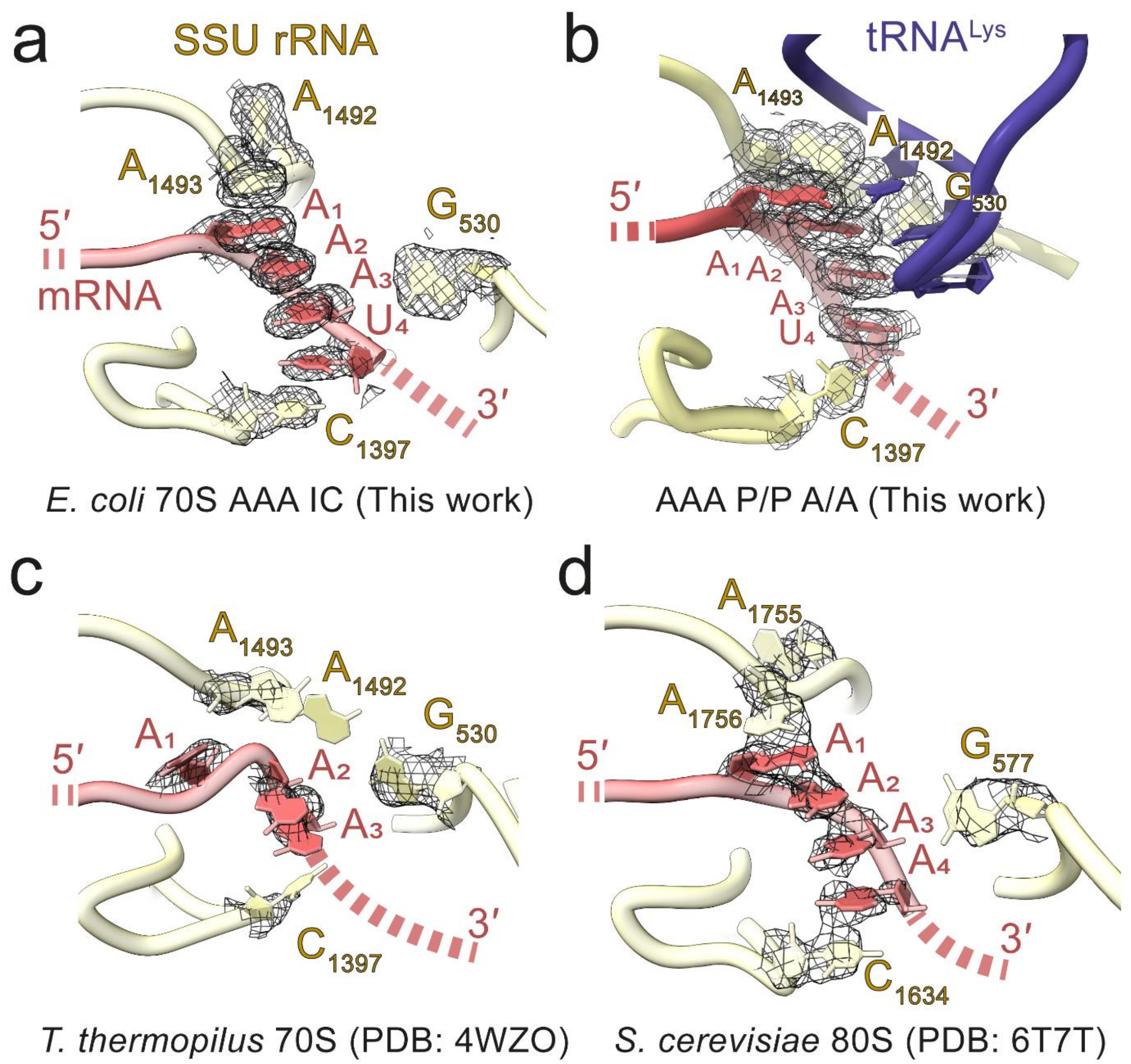

Supplementary Fig. 12. Close-up comparison of interactions between 16S rRNA (yellow) and mRNA AAA codon (red) in the A site. The nucleotide bases are represented as filled sticks and the corresponding maps are displayed as mesh. **a,** Decoding region in the structure of *E. coli* IC at 2.0 Å resolution (this paper). **b**, Decoding region in the structure of post-decoding P/P A/A at 2.3 Å resolution (this paper). **c,** The structure of IC from *T. thermophilus* determined at 3.3 Å resolution by X-ray crystallography (PDB: 4WZO [https://doi.org/10.2210/pdb4WZO/pdb]). **d,** Cryo-EM structure of 80S ribosome from *S. cerevisiae* stalled on a poly(A) track (PDB: 6T7T [https://doi.org/10.2210/pdb6T7T/pdb]) determined at 3.1 Å resolution. The mRNA A_1-A_4 sequence forms the π-stacking stabilized by yeast 18S rRNA base A1756 from the top and C1634 from the bottom. Map quality around single nucleotide bases within and around codon region is shown (each nucleotide was extracted from the structural context and flattened). The threshold level of the maps was adjusted individually.
