# Supplementary material for: Modulation of translational decoding by m6A modification of mRNA
Source: Nat Commun. 2023 Aug 8;14:4784. doi: 10.1038/s41467-023-40422-7 (PMC10409866; doi:10.1038/s41467-023-40422-7)
Supplement: Supplementary file 3 — Reporting Summary [file 41467_2023_40422_MOESM3_ESM.pdf]

nature portfolio

Corresponding author(s): Marina Rodnina & Sebastian Glatt

Last updated by author(s): Jun 18, 2023

Reporting Summary

Nature Portfolio wishes to improve the reproducibility of the work that we publish. This form provides structure for consistency and transparency in reporting. For further information on Nature Portfolio policies, see our Editorial Policies and the Editorial Policy Checklist.

Statistics

For all statistical analyses, confirm that the following items are present in the figure legend, table legend, main text, or Methods section.

n/a	Confirmed	
☐	☒	The exact sample size (*n*) for each experimental group/condition, given as a discrete number and unit of measurement
☐	☒	A statement on whether measurements were taken from distinct samples or whether the same sample was measured repeatedly
☐	☒	The statistical test(s) used AND whether they are one- or two-sided<br>*Only common tests should be described solely by name; describe more complex techniques in the Methods section.*
☒	☐	A description of all covariates tested
☐	☒	A description of any assumptions or corrections, such as tests of normality and adjustment for multiple comparisons
☐	☒	A full description of the statistical parameters including central tendency (e.g. means) or other basic estimates (e.g. regression coefficient) AND variation (e.g. standard deviation) or associated estimates of uncertainty (e.g. confidence intervals)
☐	☒	For null hypothesis testing, the test statistic (e.g. *F*, *t*, *r*) with confidence intervals, effect sizes, degrees of freedom and *P* value noted<br>*Give P values as exact values whenever suitable.*
☒	☐	For Bayesian analysis, information on the choice of priors and Markov chain Monte Carlo settings
☒	☐	For hierarchical and complex designs, identification of the appropriate level for tests and full reporting of outcomes
☒	☐	Estimates of effect sizes (e.g. Cohen's *d*, Pearson's *r*), indicating how they were calculated

Our web collection on statistics for biologists contains articles on many of the points above.

Software and code

Policy information about availability of computer code

Data collection	EPU v.2.10.0.1941REL (Thermo Fisher) for cryo-EM. Pro-Data SX (Applied Photophysics) for stopped-flow experiments. Liquid scintillation data were collected using QuantaSmart (Perkin Elmer). xCellence rt imaging software (first version) for single-molecule FRET experiments (Olympus Corporation).
Data analysis	ChimeraX 1.2.5, CryoSPARC 3.3.0, DeepEMhancer 0.14, Durchlichtelektronenmikroskopiebilddatenentzerrungwerkzeug 1.0.9, Excel 365, ISOLDE 1.4, Namdinator 2.12, Phenix 1.19.2-4158, PyMOL 1.7, Relion 3.1, WinCOOT 0.9.7 EL, vbFRET (http://vbfret.sourceforge.net/), GraphPad Prism v8. Single-molecule FRET data were analyzed using a custom script written in MATLAB (2011b, MathWorks) using the in-built statistical toolbox and is described in Adio et al., 2015, Nat Commun.

For manuscripts utilizing custom algorithms or software that are central to the research but not yet described in published literature, software must be made available to editors and reviewers. We strongly encourage code deposition in a community repository (e.g. GitHub). See the Nature Portfolio guidelines for submitting code & software for further information.

Data

Policy information about availability of data

All manuscripts must include a data availability statement. This statement should provide the following information, where applicable:

- Accession codes, unique identifiers, or web links for publicly available datasets
- A description of any restrictions on data availability
- For clinical datasets or third party data, please ensure that the statement adheres to our policy

The structure coordinates have been deposited at the Protein Data Bank (https://www.rcsb.org/) with the following PDB IDs: 8BGH (AAA IC), 8BF7 (AAA A/A P/P), 8BHP (m6AAA IC), 8BHN (m6AAA A/A P/P), 8BHL (Am6AA IC), 8BHJ (Am6AA A/A P/P), 8BGE (AAm6A IC), 8BH4 (AAm6A A/A P/P), 8BIL (AAA unreacted IC), 8BIM (AAm6A unreacted IC).
The cryo-EM maps have been deposited at the EMDB (https://www.ebi.ac.uk/emdb/) with the following IDs: EMD-16031 (AAA IC), EMD-16015 (AAA A/A P/P), EMD-16065 (m6AAA IC), EMD-16062 (m6AAA A/A P/P), EMD-16059 (Am6AA IC), EMD-16057 (Am6AA A/A P/P), EMD-16029 (AAm6A IC), EMD-16047 (AAm6A A/A P/P), EMD-16081 (AAA unreacted IC), EMD-16082 (AAm6A unreacted IC).
Original micrographs have been deposited to EMPIAR (https://www.ebi.ac.uk/empiar/) with the following IDs: 11287 (AAA), 11290 (m6AAA), 11289 (Am6AA), 11288 (AAm6A), 11291 (unreacted AAA), 11292 (unreacted AAm6A).
Ensemble kinetics data are provided in the Source Data file. Processed smFRET data are provided in the Source Data file. Original images of smFRET experiments are available upon request due to their large size and the lack of any relevant public database.

Research involving human participants, their data, or biological material

Policy information about studies with human participants or human data. See also policy information about sex, gender (identity/presentation), and sexual orientation and race, ethnicity and racism.

Reporting on sex and gender	n/a
Reporting on race, ethnicity, or other socially relevant groupings	n/a
Population characteristics	n/a
Recruitment	n/a
Ethics oversight	n/a

Note that full information on the approval of the study protocol must also be provided in the manuscript.

Field-specific reporting

Please select the one below that is the best fit for your research. If you are not sure, read the appropriate sections before making your selection.

☒ Life sciences ☐ Behavioural & social sciences ☐ Ecological, evolutionary & environmental sciences

For a reference copy of the document with all sections, see nature.com/documents/nr-reporting-summary-flat.pdf

Life sciences study design

All studies must disclose on these points even when the disclosure is negative.

Sample size	No specific statistical method was used to determine sample size. For cryo-EM analyses, details of the sample size are listed in the Supplementary Table 1 ans was chosen so that high resolution structures are constructed. The number of data points for each technical replicate in the stopped-flow is 4000, which is default setting of the stopped-flow apparatus and is sufficient to ensure precision of the fir <1%. The sample size for single-molecule FRET was determined by analysis of 3000 to 10000 spots showing Cy3 and Cy5 fluorescence. The sample size is sufficient to ensure that the R-squared values of the exponential fits are larger than 0.97 throughout the study.
Data exclusions	For cryo-EM analysis, standard 2D and 3D classification protocols were performed to identify optimal particle sets and exclude suboptimal particles. No data were excluded in the ensemble kinetics experiments. For single-molecule FRET experiments, traces that did not show Cy3 or Cy5 fluorescence, did not show characteristic anticorrelation of Cy3 and Cy5 and the single-step photobleaching were excluded from further analysis.
Replication	All biochemical and single-molecule FRET experiments were repeated as independent experiments, as indicated in the figure legends.
Randomization	No specific randomization was used in this study. Randomization is not relevant for this study because the experiments did not require allocation of individuals into groups.

Blinding	No specific blinding was used in this study. Blinding is not relevant for this study because no information of the experiments had the potential to influence the results.

Reporting for specific materials, systems and methods

We require information from authors about some types of materials, experimental systems and methods used in many studies. Here, indicate whether each material, system or method listed is relevant to your study. If you are not sure if a list item applies to your research, read the appropriate section before selecting a response.

Materials & experimental systems

n/a	Involved in the study
☒	☐ Antibodies
☒	☐ Eukaryotic cell lines
☒	☐ Palaeontology and archaeology
☒	☐ Animals and other organisms
☒	☐ Clinical data
☒	☐ Dual use research of concern
☒	☐ Plants

Methods

n/a	Involved in the study
☒	☐ ChIP-seq
☒	☐ Flow cytometry
☒	☐ MRI-based neuroimaging
